# Implementing Precision Psychiatry: A Systematic Review of Individualized Prediction Models for Clinical Practice

**DOI:** 10.1093/schbul/sbaa120

**Published:** 2020-09-11

**Authors:** Gonzalo Salazar de Pablo, Erich Studerus, Julio Vaquerizo-Serrano, Jessica Irving, Ana Catalan, Dominic Oliver, Helen Baldwin, Andrea Danese, Seena Fazel, Ewout W Steyerberg, Daniel Stahl, Paolo Fusar-Poli

**Affiliations:** 1 Early Psychosis: Interventions and Clinical-detection Lab, Department of Psychosis Studies, Institute of Psychiatry, Psychology and Neuroscience, King’s College London, 16 De Crespigny Park, London, UK; 2 Institute of Psychiatry and Mental Health, Department of Child and Adolescent Psychiatry, Hospital General Universitario Gregorio Marañón School of Medicine, Universidad Complutense, Instituto de Investigación Sanitaria Gregorio Marañón, CIBERSAM, Madrid, Spain; 3 Department of Child and Adolescent Psychiatry, Institute of Psychiatry, Psychology and Neuroscience, King’s College London, London, UK; 4 Division of Personality and Developmental Psychology, Department of Psychology, University of Basel, Basel, Switzerland; 5 Biostatistics Department, Institute of Psychiatry, Psychology and Neuroscience, King’s College London, London, UK; 6 Department of Psychiatry, Basurto University Hospital, Bilbao, Spain; 7 Mental Health Group, BioCruces Health Research Institute, Bizkaia, Spain; 8 Neuroscience Department, University of the Basque Country UPV/EHU, Leioa, Spain; 9 Social, Genetic and Developmental Psychiatry Centre, King’s College London, London, UK; 10 National and Specialist CAMHS Clinic for Trauma, Anxiety, and Depression, South London and Maudsley NHS Foundation Trust, London, UK; 11 Department of Psychiatry, University of Oxford, Oxford, UK; 12 Department of Biomedical Data Sciences, Leiden University Medical Centre, Leiden, the Netherlands; 13 Department of Public Health, Erasmus MC, Rotterdam, the Netherlands; 14 OASIS Service, South London and Maudsley NHS Foundation Trust, London, UK; 15 Department of Brain and Behavioral Sciences, University of Pavia, Pavia, Italy; 16 National Institute for Health Research, Maudsley Biomedical Research Centre, South London and Maudsley NHS Foundation Trust, London, UK

**Keywords:** risk, prognosis, prediction, individualized, prevention, evidence, implementation, validation

## Abstract

**Background:**

The impact of precision psychiatry for clinical practice has not been systematically appraised. This study aims to provide a comprehensive review of validated prediction models to estimate the individual risk of being affected with a condition (diagnostic), developing outcomes (prognostic), or responding to treatments (predictive) in mental disorders.

**Methods:**

PRISMA/RIGHT/CHARMS-compliant systematic review of the Web of Science, Cochrane Central Register of Reviews, and Ovid/PsycINFO databases from inception until July 21, 2019 (PROSPERO CRD42019155713) to identify diagnostic/prognostic/predictive prediction studies that reported individualized estimates in psychiatry and that were internally or externally validated or implemented. Random effect meta-regression analyses addressed the impact of several factors on the accuracy of prediction models.

**Findings:**

Literature search identified 584 prediction modeling studies, of which 89 were included. 10.4% of the total studies included prediction models internally validated (*n* = 61), 4.6% models externally validated (*n* = 27), and 0.2% (*n* = 1) models considered for implementation. Across validated prediction modeling studies (*n* = 88), 18.2% were diagnostic, 68.2% prognostic, and 13.6% predictive. The most frequently investigated condition was psychosis (36.4%), and the most frequently employed predictors clinical (69.5%). Unimodal compared to multimodal models (β = .29, *P* = .03) and diagnostic compared to prognostic (β = .84, *p* < .0001) and predictive (β = .87, *P* = .002) models were associated with increased accuracy.

**Interpretation:**

To date, several validated prediction models are available to support the diagnosis and prognosis of psychiatric conditions, in particular, psychosis, or to predict treatment response. Advancements of knowledge are limited by the lack of implementation research in real-world clinical practice. A new generation of implementation research is required to address this translational gap.

## Introduction

Precision medicine is an emerging approach for disease prevention, diagnosis, and treatment that considers individual variability in patient and disease characteristics, genes, environment, and lifestyle of each person.^1,2^ The concept of precision medicine is not new; clinicians have been working to personalize care tailored to people’s individual health needs throughout the history of medicine (eg, matching human blood groups across donors and recipients during blood transfusion).^3^ Yet, modern advancements of knowledge in the field of individualized prediction modeling have allowed the consolidation of an evidence-based science of precision medicine.^4^ Prediction modeling can be used to forecast the probability of a certain condition being present (diagnostic models), outcomes (prognostic models), or the response to interventions (predictive models) at the individual subject level. From a methodological perspective, individualized prediction modeling research includes studies that investigate the development, internal or external validation of prediction models, and prediction model impact studies, which investigate the real-world effect of using prediction models in clinical practice.^5^ External validity is the extent to which the predictions can be generalized to the data from plausibly related settings, while internal validity is the extent to which the predictions fit the derivation data after controlling for overfitting and optimism, with the latter representing the difference in a model’s performance in the derivation data and unseen individuals (for further details see^4^).

More recently, individualized prediction models have been developed in psychiatry,^4^ and a new field of precision psychiatry has emerged.^6–8^ The area where individualized prediction models have been more extensively investigated in psychiatry relates to psychotic disorders. The high personal, clinical, and societal burden associated with psychosis, coupled with the limited pathophysiological understanding, has stimulated research into diagnostic prediction models. Incorporation of a clinical staging model for psychosis,^9^ together with the emergence of the clinical high-risk state for psychosis (CHR-P),^10,11^ has prompted research into prognostic prediction models, as well as several ongoing international collaborations.^12^ The associated need to stratify or personalize early intervention or preventive treatment for psychosis^13,14^ has stimulated research of predictive prediction models. Furthermore, emerging research has indicated that prediction modeling can benefit from transdiagnostic approaches that allow methodological cross-fertilization across other nonpsychotic disorders.^15–17^

Despite the increasing number of records published in this area over recent years, the impact of precision psychiatry for psychosis, and more broadly for clinical practice, is unclear. No study to our knowledge has comprehensively reviewed the advancements and challenges of prediction modeling in clinical psychiatry to date. Our primary aim was to systematically appraise diagnostic, prognostic, or predictive individualized prediction models that can be considered for clinical use in psychiatry, with a specific focus on psychosis; the secondary aim was to test potential moderating factors. The evidence reviewed was then used to formulate pragmatic recommendations to advance knowledge in this area. To address the potential impact of precision psychiatry, we focused on diagnostic, prognostic, and predictive prediction model studies with at least internal or external validation and implementation studies.

## Methods

This study (study protocol: PROSPERO CRD42019155713) was conducted in accordance with the RIGHT^18^ and PRISMA^19^ statements (supplementary table 1).

### Search Strategy and Selection Criteria

A multistep independent researcher systematic literature search strategy was used to identify the relevant articles. First, the Web of Science, Cochrane Central Register of Reviews, and Ovid/ PsycINFO database were searched, from inception until July 21, 2019 in English (specific search terms are reported in supplementary methods 1). Second, the references of the articles identified in previous reviews in the field and the references from the included studies were manually searched to identify additional relevant records. Abstracts identified through the previous step were then screened and, after the exclusion of those not relevant to the current study, their full texts were assessed against the inclusion and exclusion criteria. In a fourth step, a researcher with expertise in risk estimation models in psychiatry (E.S.) further checked the articles against the core biostatistical inclusion criteria (ie, presence of appropriate internal or external validation).

The inclusion criteria were (1) original studies or study protocols published in the databases searched or gray literature; (2) studies reporting the diagnostic (principally predicts the presence of a certain condition), prognostic (principally predicts the clinical outcomes in the absence of therapy^20^), predictive (principally predicts the response to a particular intervention^20^), or implementation of risk estimation models; (3) providing estimates at the individual subject level or in subgroups; (4) studies investigating individuals affected by mental disorders or mental conditions or individuals at risk of mental disorders, defined according to established psychometric criteria, and (5) diagnostic, prognostic, or predictive studies that performed at least a proper internal or external validation (see below). The exclusion criteria were: (1) abstracts, conference proceedings, reviews, or meta-analyses; (2) diagnostic, prognostic, or predictive models that did not provide individualized or subgroup risk estimates; (3) diagnostic, prognostic, or predictive studies that did not perform any proper internal or external validation (see supplementary methods 2); or (4) predictors-finding studies that did not report prediction models.

### Descriptive Measures and Data Extraction

The variables extracted in the current review included items listed in the “Checklist for critical Appraisal and data extraction for systematic Reviews of prediction Modelling Studies” (CHARMS^21^). Additional variables were included^22^ as detailed in the supplementary methods 3. When more than one outcome per study was found in the same category, we extracted the information for the primary outcome, as defined in each article, unless the study reported multiple primary co-outcomes.

### Quality Assessment

Risk of bias was assessed for each of the included studies adapting “The Prediction Model Risk of Bias Assessment Tool” (PROBAST v5/05/2019^5,23^). PROBAST includes 4 steps and assesses the risk of bias and applicability of 4 core domains (participants, predictors, outcome, and analysis) to obtain an overall judgment of the risk of bias.^5^ An outcome is considered to be at high risk of bias when at least one of the questions answered is not appropriate (no or probably no). The overall risk of bias is considered high when one or more domains are considered to be at high risk^24^ (details can be found in supplementary methods 4).

### Data Analysis

All the included studies were systematically summarized in tables stratified by the model type (diagnostic, prognostic, and predictive)—those implemented were then discussed in a separate section—and reporting core descriptive variables (supplementary methods 5). The top 10% of the most widely employed predictors and all the studied conditions were summarized in graphs, and the specific methodological characteristics of the studies were summarized in a separate table. These descriptive analyses were complemented by the Pearson correlation between apparent vs external accuracy within the models that reported both.^16, 25–52^ We further conducted meta-analytical regressions to estimate the association between accuracy and (1) the type of validation (internal vs external); (2) the type of accuracy measure (area under the curve [AUC] vs C-statistics vs accuracy, with the latter category including accuracy measures other than AUC or C-statistics as defined by each study); (3) the type of model (diagnostic vs prognostic vs predictive model); (4) the number of specific predictors; (5) the type of predictors (clinical or service use or sociodemographic vs any biomarker—neuroimaging or electroencephalography or magnetoencephalography or proteomic or genetic or cognitive—vs a combination of modalities); (6) the modality of predictors (unimodal, using only 1 type of predictor, eg, clinical only, vs multimodal, using more than 1 type of predictor, eg, clinical and biomarker); (7) type of analysis (machine learning vs statistical modeling, as defined in supplementary methods 6). For analyses 4–7, we also included the interaction between accuracy and meta-regressors. For analyses 2–7, we used accuracy values prioritizing external validation over internal validation, in line with the previous meta-analyses of prediction models.^53^ In the case of multiple studies on the same prediction model in which the previous order of priority could not be applied, the study with the largest data set was employed. We performed a meta-regression of the difference between logit transformed accuracy (because of the bounded nature of AUC^53^) using a random effect meta-analysis model, taking 1–7 clustering of comparisons into account.^53^ The analyses were performed with Comprehensive Meta-Analysis Version 3.^54^

## Results

### Database

**Fig. 1.**
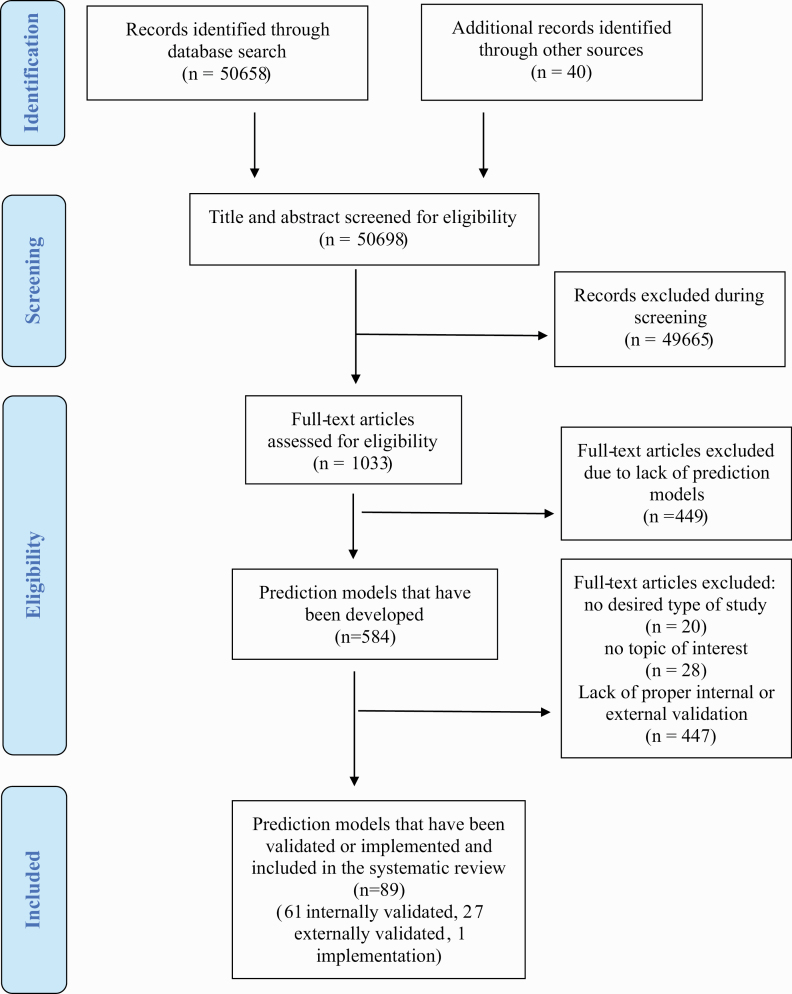
Preferred Reporting Items for Systematic Reviews and Meta-Analyses flowchart outlining study selection process.

**Fig. 2.**
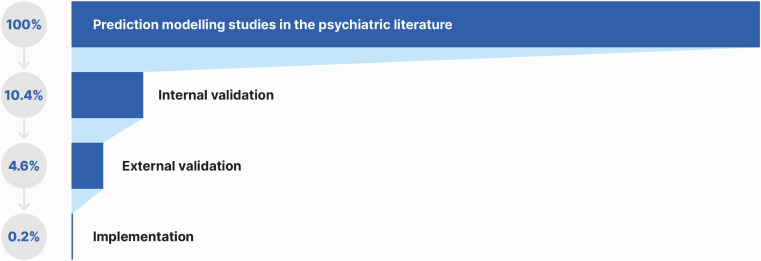
Proportion of prediction models studies developed, internally validated, externally validated, and implemented in the psychiatric literature.

**Fig. 3.**
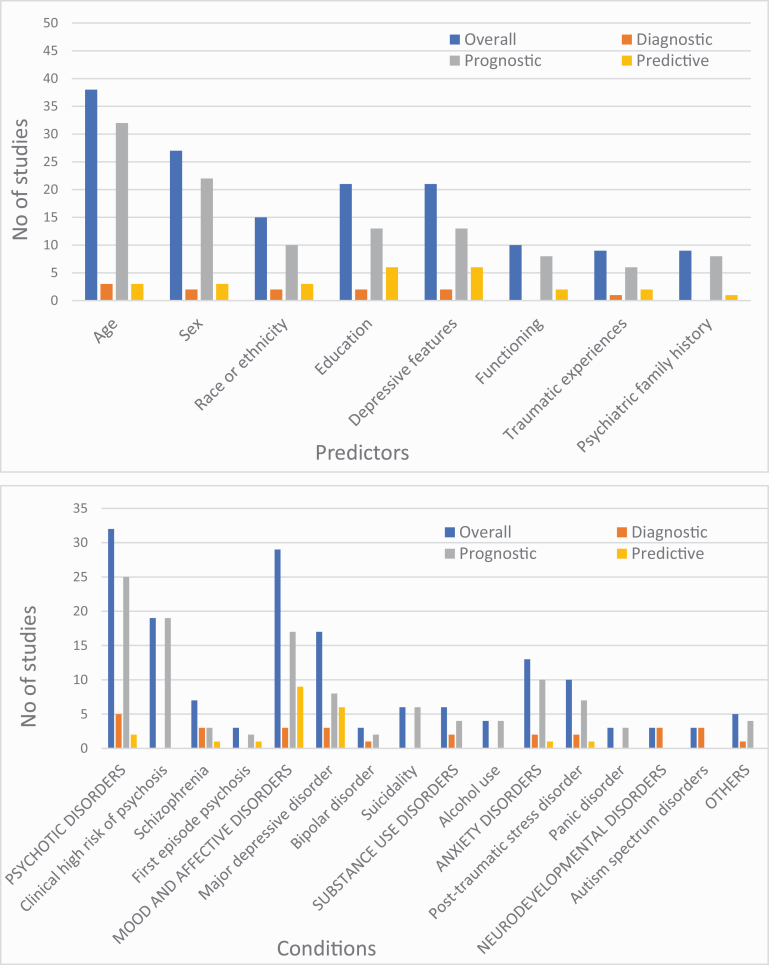
Most frequently reported predictors (above, top 10%) and conditions (below, all) in the included studies.

The literature search yielded 50 698 records and, after the exclusion of nonrelevant abstracts, 1033 full-text articles were screened to identify a total of 584 prediction studies reporting on prediction models developed. These models were then screened for eligibility against the inclusion and exclusion criteria to identify 89 studies with individualized prediction models, which were validated or implemented and represented the final sample (PRISMA; figure 1): 61 were internally validated (10.4% of the total models developed), 27 were externally validated (4.6% of the total models developed), and 1 (0.2% of the total models developed) described a protocol for the implementation of a prediction model (figure 2). Thirty point three percent (27/89) of the prediction models included were externally validated. 8.2% studies reported on diagnostic prediction models, 68.2% on prognostic models, and 13.6% on predictive models; 55.6% of studies employed sociodemographic predictors, 69.5% employed clinical predictors, 10.2% employed cognitive predictors, 13.6% employed service use predictors, 25.0% employed physical health predictors, 17.0% employed neuroimaging predictors, 0.4% employed magnetoencephalography or electroencephalography predictors, 0.1% employed proteomic data, and 2.3% employed genetic predictors. The most frequently reported predictors were age (*n* = 38, 45.8%), sex (*n* = 27, 32.5%), education (*n* = 21, 25.3%), and depressive symptoms (*n* = 18, 21.7%; figure 3). The most frequently reported condition was psychosis (36.4%; figure 3). The total sample size was 3 889 457 individuals, ranging from 29^55^ to 2 960 929^56^ individuals. The average age ranged from 1.8^57^ to 64.7^38^ years. The source of data encompassed cohorts (46 studies, 52.3%), case-control studies (13 studies, 14.8%), clinical trials (16 studies, 18.2%), and registry data (13 studies, 14.8%).

The most frequent type of external validation was geographical, examining the model performance in other centers or regions 24/27 (88.9%). Internal validation was more frequently done by cross-validation in 34/61 (55.7%). The most frequent modeling method was machine learning in 35/88 (39.8%) (supplementary table 2). In half of the studies (51.1%), there was no explicit handling of missing data; imputation (27.3%) was the most common method for data missingness (supplementary table 2). AUC was the most commonly reported measure of model performance (78.4%; supplementary table 2). Only 10.2% of the studies presented their model in full: almost half of them did not present any details of their model (46.6%) or the calibration results clearly (47.7%; supplementary table 2).

### Diagnostic Risk Estimation Models

Four studies employed neuroimaging methods^58–60^ and proteomic data^61^ to classify individuals with schizophrenia compared to healthy controls (HC)^58,59,61^ or to differentiate schizophrenia spectrum disorder and HC with or without impaired social functioning^60^ (supplementary table 3). One study employed clinical predictors to discriminate between affective and schizophrenia spectrum psychoses.^62^

Two studies employed neuroimaging to differentiate unipolar vs bipolar depression^25^ or major depression vs dysthymia in individuals with panic disorder and agoraphobia.^63^ Another study used clinical predictors to distinguish melancholic vs non-melancholic features in individuals with major depression.^64^

A neuroimaging study discriminated smokers and nonsmoking HC.^65^ Another study using sociodemographic, clinical, and cognitive data discriminated individuals with cocaine dependence from HC.^66^ Problematic internet use was discriminated from HC using clinical and sociodemographic predictors.^26^ Two studies classified posttraumatic stress disorder in veterans using sociodemographic and clinical predictors^67^ or magnetoencephalography.^68^ Three studies focused on autism spectrum disorders to discriminate them from attention deficit hyperactive disorder^69,70^ or from HC^57^ using clinical predictors^69,70^ or genomic biomarkers^57^ (supplementary table 3).

### Prognostic Models

A considerable proportion of the prognostic risk estimation studies^16, 27–32, 71–82^ (31.7%) investigated the CHR-P^83^ (supplementary table 4). These studies focused on the prediction of psychosis onset in CHR-P individuals (*n* = 13),^27–29,71–80^ functional outcomes and disability in CHR-P individuals (*n* = 2),^81,82^ psychosis onset in individuals undergoing a CHR-P assessment (pretest risk *n* = 1),^30^ and the transdiagnostic onset of psychosis in secondary mental health care (*n* = 3).^16,31,32^ Six of these studies employed sociodemographic or clinical predictors only,^16,28,31,32,73,74^ 1 employed sociodemographic and service use data,^30^ 2 included cognitive measures beyond sociodemographic and clinical data,^27,72^ 3 included cognitive measures alone,^29,71,77^ 1 employed electroencephalography predictors,^75^ 3 neuroimaging alone,^76,78,80^ and 2 neuroimaging in association with clinical measures^81,82^ or in association with sociodemographic, clinical, and cognitive measures (*n* = 1).^79^ Four other studies focused on established psychosis using different combinations of sociodemographic, clinical, service use, cognitive, and physical health predictors to forecast psychotic relapses,^84^ hospital admission,^33^ employment, education or training status,^34^ and mortality.^85^ Nine studies focused on depression.^35–40,82,86,87^ A combination of sociodemographic, clinical, and physical health factors was used by 3 studies to predict the onset of major depression in the general population^35–37^ and by 5 other studies to predict persistence^38,86,87^ or recurrence^39,40^ of major depression. A further study predicted disability in recent-onset depression using clinical and neuroimaging data.^82^ One study focused on the onset of bipolar spectrum disorders in youth at family risk using sociodemographic and clinical factors,^88^ while another one predicted cognitive impairment in bipolar disorder using sociodemographic and cognitive factors.^89^ Six studies used a combination of sociodemographic, clinical, physical health, and service use to predict suicidality, focusing on suicide ideation in the general population,^41,90^ suicide attempts after outpatient visits,^56^ suicide attempts in adolescents,^91^ suicidal behavior,^92^ or deaths by suicide after hospitalization in soldiers.^93^ Seven studies focused on posttraumatic stress disorder (PTSD).^94–100^ Three studies employed a combination of sociodemographic, clinical, physical health, and service use factors to predict the onset of PTSD^94–96^ or the remission of PTSD (*n* = 3 studies),^97–99^ and a further study used clinical predictors alone to forecast PTSD features in soldiers.^100^ Sociodemographic, clinical, and physical health data were used by 2 studies^42,43^ to predict the onset of generalized anxiety disorders and panic disorder in the general population and by another study to predict the recurrence of panic disorder.^44^

Two studies predicted alcohol use in young people using sociodemographic and clinical^45,46^ predictors in combination with cognitive^46^ predictors, while another 2 studies predicted abstinence from heavy drinking using sociodemographic and/or clinical^47,101^ data. A prediction model forecasted offending behavior in schizophrenia and delusional disorder using forensic information.^102^ Compulsory admission into psychiatric wards was predicted by a combination of sociodemographic, clinical, and service use factors,^103^ and medication-induced altered mental status in hospitalized patients was predicted by sociodemographic, clinical, service use, and physical health data.^104^ Other models predicted the onset of common mental disorder in a working population using sociodemographic, clinical, and physical health^105^ variables, mental health hospital readmission using sociodemographic, clinical, and service use^106^ data, and violent offending in severe mental disorders using sociodemographic, clinical, and service use^48^ data.

### Predictive Models

Two studies employed a combination of clinical, sociodemographic, or physical health features to predict remission^49,50^ or response to antidepressants^107,108^ in major depression. Three studies predicted the onset of treatment-resistant depression using clinical and sociodemographic variables,^51,52,109^ service use data,^52,109^ and physical health data.^109^ A study employed clinical and sociodemographic data to predict the level of functioning at 4 and 52 weeks after antipsychotic treatment in patients with first-episode psychosis.^110^ Two studies predicted the clinical response to transcranial magnetic stimulation combining neuroimaging and electroencephalography factors.^55,111^ A further study employed clinical and physical health data to predict treatment dropout from psychotherapy in anxiety disorders^112^ (supplementary table 5)

### Implementation of Prediction Models

Among externally validated models, the transdiagnostic model predicting psychosis onset in secondary mental health care,^16,31,32^ the model predicting psychosis onset in CHR-P,^27,72^ the model predicting the onset of generalized anxiety disorders and panic disorder in the general population,^42,43^ and the model predicting the onset of major depression in the general population^36,37^ were all replicated twice (table 1). None of the models included in the current systematic review were fully implemented in clinical practice. However, 1 study^113^ described the protocol for the implementation of the transdiagnostic risk calculator to detect individuals at risk of psychosis in secondary mental health care.^16,31,32^ The core aim of this study was to integrate the prediction model in the local electronic health register and evaluate the clinician’s adherence to the recommendations made by the risk calculator.^113^

**Table 1:** Replicated prediction models (all prognostic)

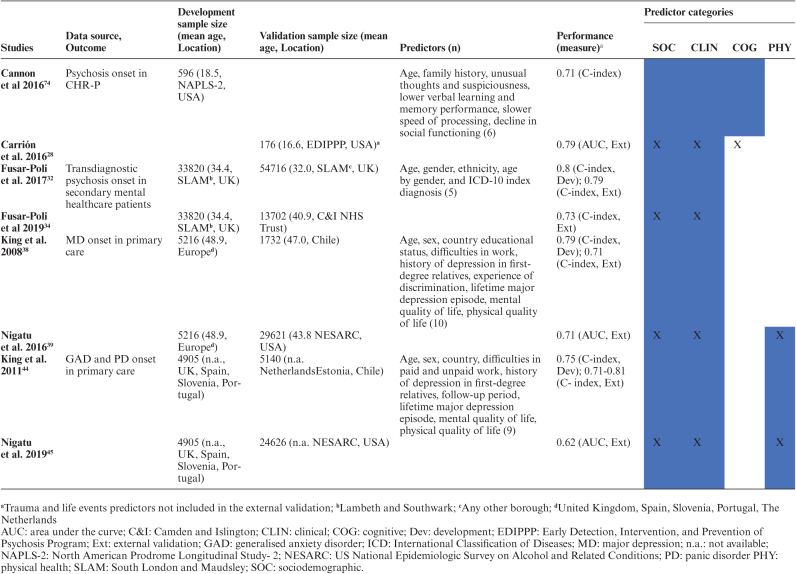

### Accuracy of Prediction Models and Meta-Regressions

**Fig. 4.**
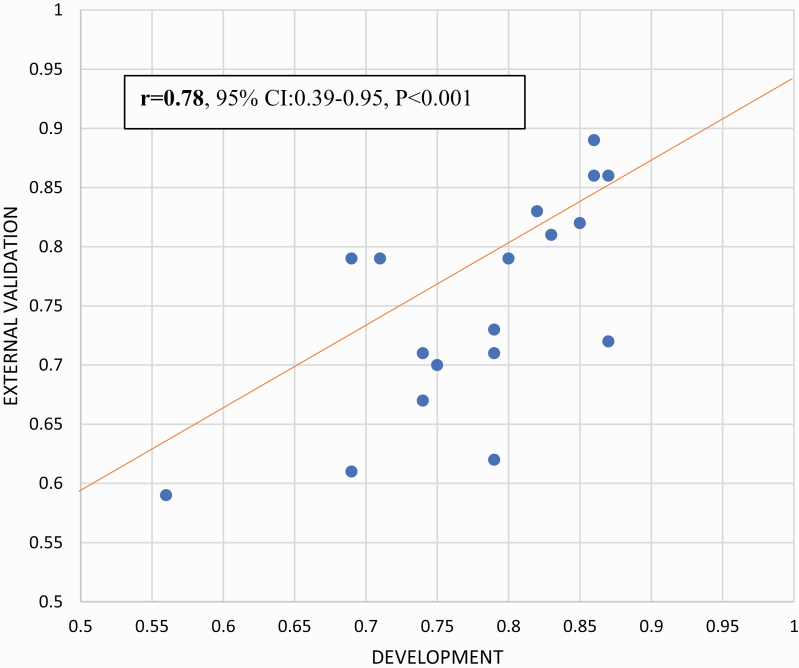
Correlation between apparent and external accuracy (*n* = 18).

Accuracy of prediction models was highly variable, ranging from 0.56^40^ to 1.0^71^ (0.69^25^–0.96^69^ for diagnostic models, 0.56^40^ to 1.0^71^ for prognostic models, and 0.66^108^ to 0.92^114^ for predictive models) (supplementary tables 3–5). Within the nonoverlapping prediction model studies that reported apparent and external accuracy (*n* = 18), the 2 measures were strongly correlated (*r* = .78, 95% CI: 0.39–0.95, *P* < .001; figure 4). Meta-regressions revealed that accuracy was higher in unimodal (*n* = 25) vs multimodal (*n* = 71) prediction models (β = .29, *P* = .03), diagnostic (*n* = 14) vs prognostic (*n* = 51; β = .84, *P* < .001) models, and diagnostic (*n* = 14) vs predictive (*n* = 11; β = .87, *P* = .002) models, but no other significant meta-regressions or interactions were detected (supplementary results and supplementary table 6).

### Quality of Prediction Models

Applying PROBAST, 94.3% of the included studies were found to be at high risk of bias. The results from the different domains were heterogeneous: 1.1% were at high risk of bias in the participants domain, 65.9% in the predictors domain, 90.9% in the outcomes domain, and 81.8% in the analysis domain (supplementary table 7; supplementary figure 1).

## Discussion

This is the first large-scale systematic review to summarize the transdiagnostic and life span-inclusive evidence regarding diagnostic, prognostic, or predictive prediction models that have been internally and externally validated and, thus, can be considered for clinical implementation in psychiatry. Currently, only 10.4% of the total models developed are internally validated, 4.6% are externally validated, and 0.2% are considered for implementation. Most of the models validated were prognostic, followed by diagnostic and more infrequently predictive models. Most research in this area focused on psychosis and was life span inclusive. Most prediction models employed clinical predictors. Many studies were at high risk of bias and accuracy was mediated by several factors.

The main finding of this study is that precision psychiatry has developed into a consolidated area of clinical research, with a substantial number of individualized prediction models developed and validated on data from 3 889 457 participants aged from 1.8 to 64 years. These substantial advancements in the field of precision psychiatry reflect a life span-inclusive approach. Several validated individualized prediction models are nowadays available, transdiagnostically targeting many psychiatric conditions encompassing psychotic disorders, affective disorders, substance use disorders, anxiety disorders, neurodevelopmental disorders, and several clinically relevant outcomes as well. However, to date, psychosis research has mostly led (36.4%) precision psychiatry. Notably, the majority (68.2%) of the current psychiatric prediction models were prognostic, with CHR-P studies representing a leading field (31.7%) in this domain (21.6% across all prognostic, diagnostic, and predictive models). This finding confirms the traction role of psychosis research, as well as the close link between precision psychiatry and preventive approaches. Psychiatry as a discipline is essentially “Hippocratic,” whereby the prediction of outcomes becomes more relevant than the ascertainment of cross-sectional diagnostic categories.^4^ The validity of diagnostic categories in psychiatry has always been criticized and it has recently been further questioned by transdiagnostic approaches, which challenged discrete and fixed self-delimitating boundaries across International Classification of Diseases or *Diagnostic and Statistical Manual of Mental Disorders* entities.^15,17^ These considerations are particularly valid for early psychosis, where the prediction of outcomes can inform treatment approaches and can explain why diagnostic models were not so frequent (18.2%). Predictive models were even less frequently investigated (13.6%), presumably because these types of studies are inherently more complex to run owing to the intervention-related component. Despite these speculations, accuracy in diagnostic models remained superior to prognostic and predictive models, presumably because diagnostic models rely on more established gold standards to define outcomes.

Despite the substantial progress in developing and validating individualized prediction models for psychiatry, this study also highlighted some important barriers to the advancement of knowledge. The first barrier is that, across the overall pool of prediction models developed and published in the broader psychiatric literature (*n* = 584), we found only about 15% (*n* = 88) to be properly validated (*n* = 61: 10.4% internal validation and *n* = 27: 4.6% independent external validation). Within those included in the review, about one-third were validated in external databases (supplementary limitations). This finding aligns with a previous review suggesting that external validation of prediction models is infrequent.^115^ A growing body of evidence has confirmed a replicability crisis in several areas of scientific knowledge, such as cancer research,^116^ economics,^117^ behavioral ecology economics,^117^ and genetic behavior research.^118^ Since precision psychiatry is a relatively emerging paradigm compared to other precision medicine approaches, research to date may have prioritized the development of new models over the external validation of models already established. For example, systematic reviews in chronic obstructive pulmonary disease identified a similar number of prediction models with internal (*n* = 100) and external (*n* = 38) validation to the ones reported here.^24^ However, several of these models were externally validated between 5 and 17 times.^24^ The next generation of prediction modeling in psychiatry should, therefore, consider, along with the development of new prediction models, the replication of existing algorithms across different scenarios. This would necessitate collaborative data-sharing efforts to reach critical mass (studies’ sample size ranged from 29^55^ to 2 960 929^56^ individuals) and the establishment of international clinical research infrastructures, as well as specific support from funders and stakeholders. The current study should also educate editors and reviewers who too often devalue replication studies because they feel that these studies have limited advancement of knowledge compared to the original publications. In reality, focusing on the reproducibility of existing prediction models and updating existing prognostic models, as opposed to dropping these models and developing new ones from scratch, is the recommended procedure to maximize the efficiency of research.^4^

This study also provides relevant methodological evidence. For example, to date, most models (69.5%) are based on clinical predictors and there is no evidence that more complex models encompassing biomarkers or a large number of predictors (which may be more prone to overfitting issues) or advanced analytical methods, such as machine learning, outperform other types of prediction models. These findings align with recent studies indicating that complex machine learning models do not outperform more parsimonious clinically based models developed through standard statistical approaches.^53,119^ The current study adds further methodological value by showing that, in psychiatry, for a given apparent accuracy (we found no difference across various accuracy measures), the expected external accuracy can be estimated with a correlating factor of .78 (95% CI: 0.39–0.95; figure 4). Editors and reviewers can use this factor to assess the external accuracy of prediction models that have not been internally/externally validated. However, current guidelines recommend performing at least internal validation,^4^ which, if properly performed, can accurately index the true external generalizability of the model (as shown in our meta-regressions).

An associated problem is that 94.3% studies included in the current review—which adopted stringent inclusion criteria focusing on validated studies—were eventually classified at high risk of bias, mostly because of the high risk of bias in the outcomes and analysis domain. These biases may potentially be even more substantial in the wider literature, limiting the implementation of precision psychiatry. Although the PROBAST threshold for this bias may be too strict, our findings are consistent with an independent review, which applied PROBAST and found that 98.3% of the prediction models were at high risk of bias.^24^ Facilitating the external validation of individualized prediction models is also the most robust approach to address the currently largest barrier for precision psychiatry: real-world implementation.

The current systematic review identified only one implementation study, corresponding to 0.2% of the total pool of models developed and published, which did not report data but only described the research protocol of an ongoing project^120^ (the full implementation results have been published upon completion of our literature review).^121,122^ At the moment, precision psychiatry is severely limited by a translational gap. The implementation pathways of precision psychiatry is a perilous journey,^123^ complicated by obstacles related to patients (eg, making their data available or accepting the outputs of the risk calculator), clinicians (eg, adherence to the recommendations made by prediction models and communicating risks), providers (eg, confidentiality and accessibility of data and interpretability and utility of outputs), and funders and organizations (implementing an infrastructure enabling standard prediction procedures). Because of these challenges, most prediction models that are validated are then lost in the dearth of real-world implementation science, even for psychosis research. Implementation science itself, although much needed, is contested and complex, with the unpredictable use of results from routine clinical practice.^124,125^ For example, the Consolidated Framework for Implementation Research (CFIR)^30^ is rather theoretical^124^ and does not offer specific pragmatic guidance to precision psychiatry. A recent systematic review concluded that only 6% of studies acknowledging the CFIR used the CFIR in a meaningful way.^126^ Thus, the paucity of implementation studies of individualized prediction models in psychiatry can be secondary to the lack of a general implementation framework and practical guidance. The next generation of empirical research in the field of prediction modeling in psychiatry and psychosis research should primarily aim at filling in the implementation gap by developing a coherent and practical implementation framework, methodological infrastructures, and international implementation infrastructures.

## Conclusions

To date, several validated prediction models are available to support the diagnosis and prognosis of psychiatric conditions, in particular, psychotic disorders, or to predict the response to treatments. Advancements of knowledge are mostly limited by the limited replication and lack of implementation research in real-world clinical practice. The next generation of precision psychiatry research is required to address this translational gap.

## Supplementary Material

sbaa120_suppl_Supplementary_Materials
