# Supplementary material for: Implementing Precision Psychiatry: A Systematic Review of Individualized Prediction Models for Clinical Practice
Source: Schizophr Bull. 2020 Sep 11;47(2):284–97. doi: 10.1093/schbul/sbaa120 (PMC7965077; doi:10.1093/schbul/sbaa120)
Supplement: sbaa120_suppl_Supplementary_Materials [file sbaa120_suppl_supplementary_materials.docx]

**SUPPLEMENTARY MATERIAL**

**eTable 1, p2-3:** PRISMA statement and checklist.

**eTable 2, p4:** Methodological characteristics of individualised prediction models for clinical psychiatry

**eTable 3, p5:** Characteristics of diagnostic risk estimation models

**eTable 4, p6-9:** Characteristics of prognostic risk estimation models

**eTable 5, p10:** Characteristics of predictive risk estimation models

**eTable 6, p11:** Meta-regressions between accuracy and different moderating factors

**eTable 7, p12-13:** Quality assessment results

**eMethods 1, p14:** Search terms used in the literature search

**eMethods 2, p15:** Exclusion of models according to validation procedures

**eMethods 3, p16:** Variables included in the study

**eMethods 4, p17:** Quality assessment and risk of bias items (PROBAST v5/05/2019)

**eMethods 5, p18:** Core descriptive variables reported in the summary tables

**eMethods 6, p19:** Categories and coding of machine learning and statistical learning

**eResults, p20:** Meta-regression analyses

**eLimitations, p21:** Limitations of the current study

**eFigure 1, p22:** Risk of bias among retrieved studies

**This supplementary material has been provided by the authors to give readers additional information about their work.**

**eTable 1. PRISMA statement and checklist**

| PRISMA guidelines for meta-analysis and systematic reviews | | | |
| --- | --- | --- | --- |
| **Section/topic** | **#** | **Checklist item** | **Page** |
| **TITLE** | | |  |
| Title | 1 | Identify the report as a systematic review, meta-analysis, or both. | 1 |
| **ABSTRACT** | | |  |
| Structured summary | 2 | Provide a structured summary including, as applicable: background; objectives; data sources; study eligibility criteria, participants, and interventions; study appraisal and synthesis methods; results; limitations; conclusions and implications of key findings; systematic review registration number. | 1 |
| **INTRODUCTION** | | |  |
| Rationale | 3 | Describe the rationale for the review in the context of what is already known. | 1-2 |
| Objectives | 4 | Provide an explicit statement of questions being addressed with reference to participants, interventions, comparisons, outcomes, and study design (PICOS). | 2 |
| **METHODS** | | |  |
| Protocol and registration | 5 | Indicate if a review protocol exists, if and where it can be accessed (e.g., Web address), and, if available, provide registration information including registration number. | 2 |
| Eligibility criteria | 6 | Specify study characteristics (e.g., PICOS, length of follow-up) and report characteristics (e.g., years considered, language, publication status) used as criteria for eligibility, giving rationale. | 2 |
| Information sources | 7 | Describe all information sources (e.g., databases with dates of coverage, contact with study authors to identify additional studies) in the search and date last searched. | 2 |
| Search | 8 | Present full electronic search strategy for at least one database, including any limits used, such that it could be repeated. | e13 |
| Study selection | 9 | State the process for selecting studies (i.e., screening, eligibility, included in systematic review, and, if applicable, included in the meta-analysis). | 2 |
| Data collection process | 10 | Describe method of data extraction from reports (e.g., piloted forms, independently, in duplicate) and any processes for obtaining and confirming data from investigators. | 2 |
| Data items | 11 | List and define all variables for which data were sought (e.g., PICOS, funding sources) and any assumptions and simplifications made. | 3, e16 |
| Risk of bias in individual studies | 12 | Describe methods used for assessing risk of bias of individual studies (including specification of whether this was done at the study or outcome level), and how this information is to be used in any data synthesis. | 3, e17 |
| Summary measures | 13 | State the principal summary measures (e.g., risk ratio, difference in means). | 3 |
| Synthesis of results | 14 | Describe the methods of handling data and combining results of studies, if done, including measures of consistency (e.g., I2) for each meta-analysis. | 3 |
| Risk of bias across studies | 15 | Specify any assessment of risk of bias that may affect the cumulative evidence. | 3, e17, e22 |
| Additional analyses | 16 | Describe methods of additional analyses (e.g., sensitivity or subgroup analyses, meta-regression), if done, indicating which were pre-specified. | 3 |
| **RESULTS** |  |  |  |
| Study selection | 17 | Give numbers of studies screened, assessed for eligibility, and included in the review, with reasons for exclusions at each stage, ideally with a flow diagram. | 3 |
| Study characteristics | 18 | For each study, present characteristics for which data were extracted (e.g., study size, PICOS, follow-up period) and provide the citations. | e5-10, 7 |
| Risk of bias within studies | 19 | Present data on risk of bias of each study and, if available, any outcome level assessment (see item 12). | e12-13 |
| Results of individual studies | 20 | For all outcomes considered (benefits or harms), present, for each study: (a) simple summary data for each intervention group (b) effect estimates and confidence intervals, ideally with a forest plot. | e5-11, e20 |
| Synthesis of results | 21 | Present results of each meta-regression done, including confidence intervals and measures of consistency. | 3-8, fig2-4, e20 |
| Risk of bias across studies | 22 | Present results of any assessment of risk of bias across studies (see Item 15). | 8, e12-13, e22 |
| Additional analysis | 23 | Give results of additional analyses, if done (e.g., sensitivity or subgroup analyses, meta-regression [see Item 16]). | 6-8, fig2-4, e20 |
| **DISCUSSION** |  |  |  |
| Summary of evidence | 24 | Summarize the main findings including the strength of evidence for each main outcome; consider their relevance to key groups (e.g., healthcare providers, users, and policy makers). | 8-10 |
| Limitations | 25 | Discuss limitations at study and outcome level (e.g., risk of bias), and at review-level (e.g., incomplete retrieval of identified research, reporting bias). | 9-10, e21 |
| Conclusions | 26 | Provide a general interpretation of the results in the context of other evidence, and implications for future research. | 10 |
| **FUNDING** |  |  |  |
| Funding | 27 | Describe sources of funding for the systematic review and other support; role of funders for the systematic review. | 10 |

**eTable 2** Methodological characteristics of individualised prediction models for clinical psychiatry

|  | **Diagnostic, n (%)** | **Prognostic, n (%)** | **Predictive, n (%)** | **Overall, n (%)** |
| --- | --- | --- | --- | --- |
| **Validation** | | | | |
| Internal: Sample split | 2 (12.5) | 11 (18.3) | 0 (0.0) | 13 (14.8) |
| Internal: Cross-validation | 11 (68.7) | 19 (31.7) | 4 (33.3) | 34 (38.6) |
| Internal: Combination of methods | 1 (6.2) | 9 (15.0) | 4 (33.3) | 14 (15.9) |
| External validation | 2 (12.5)^a^ | 21 (35.0)^a^ | 4 (33.3)^a^ | 27 (30.7)^a^ |
| **Modelling method** | | | | |
| Cox hazard model | 0 (0.0) | 6 (10.0) | 0 (0.0) | 6 (8.6) |
| Logistic regression model | 4 (25.0) | 19 (31.7) | 3 (25.0) | 26 (29.5) |
| Linear regression model | 1 (6.2) | 1 (1.7) | 1 (8.3) | 3 (3.4) |
| Negative binomial model | 0 (0.0) | 0 (0.0) | 0 (0.0) | 0 (0.0) |
| Generalised linear model | 1 (6.2) | 1 (1.7) | 0 (0.0) | 2 (2.3) |
| Weibull regression model | 0 (0.0) | 0 (0.0) | 0 (0.0) | 0 (0.0) |
| Other statistical modelling methods | 0 (0.0) | 4 (6.7) | 1 (8.3) | 5 (5.6) |
| Machine learning | 8 (50.0) | 22 (36.7) | 5 (41.7) | 35 (39.8) |
| More than one method | 1 (6.2) | 5 (8.3) | 2 (16.7) | 8 (9.1)^b^ |
| None | 1 (6.2) | 2 (3.3) | 0 (0.0) | 3 (3.4) |
| **Handling of missing data** | | | | |
| Imputation | 2 (12.3) | 19 (31.7) | 3 (25.0) | 24 (27.3) |
| No missing values | 0 (0.0) | 0 (0.0) | 0 (0.0) | 0 (0.0) |
| Other methods | 1 (6.2) | 1 (1.7) | 1 (8.3) | 3 (3.4) |
| More than one method | 0 (0.0) | 3 (5.0) | 0 (0.0) | 3 (3.4) |
| Exclusion of patients or variables | 3 (18.7) | 6 (10.0) | 4 (33.3) | 13 (14.8) |
| None | 10 (62.5) | 31 (51.7) | 4 (33.3) | 45 (51.1) |
| **Model discrimination** | | | | |
| Accuracy | 4 (25.0) | 8 (13.3) | 4 (33.3) | 16 (18.2) |
| AUC | 11 (68.6) | 31 (51.7) | 7 (58.3) | 49 (55.7) |
| c-index | 0 (0.0) | 20 (33.3) | 0 (0.0) | 20 (22.7) |
| None of the above | 1 (6.2) | 1 (1.7) | 1 (8.3) | 3 (3.4) |
| **Model presentation** | | | | |
| Full equation | 2 (12.3) | 6 (10.0) | 1 (8.3) | 9 (10.2) |
| Sum score one method | 1 (6.2) | 4 (6.7) | 1 (8.3) | 6 (6.8) |
| Decision tree | 2 (12.3) | 4 (6.7) | 1 (8.3) | 7 (8.0) |
| Nomogram | 0 (0.0) | 2 (3.3) | 0 (0.0) | 2 (2.3) |
| Risk chart | 2 (12.3) | 4 (6.7) | 1 (8.3) | 7 (8.0) |
| More than one method | 4 (25.0) | 4 (6.7) | 2 (16.7) | 10 (11.4) |
| Other method | 1 (6.2) | 3 (5.0) | 2 (16.7) | 6 (6.8) |
| None | 4 (25.0) | 33 (55.0) | 4 (33.3) | 41 (46.6) |
| **Model calibration** | | | | |
| Hosmer-Lemeshow test | 0 (0.0) | 7 (11.7) | 1 (8.3) | 8 (9.1) |
| Calibration plot | 2 (12.3) | 9 (15.0) | 3 (25.0) | 14 (15.9) |
| More than one method | 1 (6.2) | 15 (25.0) | 3 (25.0) | 19 (21.6) |
| Other | 1 (6.2) | 3 (5.0) | 1 (8.3) | 5 (5.7) |
| None | 0 (0.0) | 0 (0.0) | 0 (0.0) | 0 (0.0) |
| Unclear | 12 (75.0) | 26 (43.3) | 4 (33.3) | 42 (47.7) |

^a^All studies were externally validated.

^b^6 studies combined statistical modelling and machine learning modelling technique.

**eTable 3. Characteristics of diagnostic risk estimation models**

| **Author** | **Outcome** | **Data source** | **Mean age in years (SD or range), % female** | **Sample size:**  **total** | **Predictors:**  **n: categories** | **Validation type (Dev / Ext)^a^, Main analysis** | **Performance (measure)**  **(Dev; Ext)** ^a^ | **Se/ Sp** | **PPV/ PNV** |
| --- | --- | --- | --- | --- | --- | --- | --- | --- | --- |
| **Kalmady et al. 2019^1^** | SCZ vs HC | Case-control | 30 (5.9), 35.1 | 174 | N.a: Neuroimaging | I (10-FCV), ML | 0.87 (accuracy) | 0.80; 0.93 | N.a. |
| **Rozycki et al. 2018^2^** | SCZ vs HC | Case-control | 34.6 (11.3), 42.7 | 835 | N.a: Neuroimaging | I (LSSO-CV), ML | 0.73-0.91(AUC) | N.a. | N.a. |
| **Cooper et al. 2019^3^** | SCZ vs HC | Case-control | 31.4 (10.5), 46.7 | 160 | 22: Proteomic | I (10-FCV), S | 0.81-0.92 (AUC) | N.a. | N.a. |
| **Viviano et al. 2018^4^** | Cognitive performance and social function in SZD-SD vs HC | Case-control | 33 (10.2), 37 | 263 | N.a: Neuroimaging | I (10-FCV), ML | 0.88 (AUC) | N.a. | N.a. |
| **Jauhar et al. 2018^5^** | ASP vs SSP in psychosis | Cohort | n.a (16-69), 50.6 | 202 | 20: Clinical | I (nested CV), ML | 0.89 (AUC) | 0.77-0.88/N.a. | 0.82-0.85/n.a. |
| **Redlich et al. 2014^6^** | Unipolar MD vs BD | Case-control | 37.6 (10.5), 63.8 | 174 | N.a: Neuroimaging | E (geographical) (87/ 87), ML | 0.69 (accuracy, Dev); 0.79 (accuracy, Ext) | 0.69-0.76/ 0.59-0.72 | N.a. |
| **Lueken et al. 2015^7^** | MD and/or dysthymia in PD/AG | Clinical trial | 36.7 (10.4), 67.8 | 59 | N,a: Neuroimaging | I (LSSO-CV), ML | 0.73 (accuracy) | 0,77/0,7 | N.a. |
| **Parker et al. 2015^8^** | Melancholic MD vs. non-melancholic MD | Cohort | 42.6 (13.2), 54 | 364 | 5: Clinical | I (temporal), S | None | 0.93/0,82 | N.a. |
| **Ding et al. 2017^9^** | Smokers^b^ vs non-smokers in HC | Case-control | 31.3 (9.7), 47 | 200 | N.a: Neuroimaging | I (LSSO-CV), ML | 0.73-0.75 (accuracy) | 0,76-0.79/ 0,68-0.75 | 0.7-0.75/N.a. |
| **Ahn et al. 2016^10^** | Cocaine dependence vs HC | Case-control | 40.3 (11.9), 47.5 | 54 | 6: Sociodemographic, clinical, cognitive | I (LLSO-CV), ML | 0.91 (AUC) | N.a. | N.a. |
| **Ioannidis et al. 2016^11^** | Problematic internet use^c^ vs HC | Cohort | 30.1, 34.4 | 2006 | N.a: Sociodemographic, clinical | E (geographical), ML | 0.84 (AUC, Ext) | 0.59-0.7/ 0.91-0.93. | 0.24-0.44/ 0.97-0.99 |
| **Harrington et al. 2019^12^** | PTSD vs HC in veterans | Registry data | 64.0 (15.4), 5.6 | 485 | 11: Sociodemographic, clinical | I (10-CV), S | 0.95 (AUC) | 0.99/0.99 | 0.95/1.00 |
| **James et al. 2015^13^** | PTSD vs HC in veterans | Case-control | 54 (16), 10.6 | 432 | N.a: Magnetoencephalography | I (CV), S | 0.9 (AUC) | 0.67-0.99/ 0.72-0.95 | N.a. |
| **Duda et al. 2016^14^** | ASD vs. ADHD | Cohort | n.a, 16.7 | 2925 | N.a.: Clinical | I (10-FCV), ML | 0.96 (AUC) | N.a. | N.a. |
| **Duda et al. 2017^15^** | ASD vs. ADHD | Cohort | 9.3 (3.9), 26.6 | 422 | 15: Clinical | I (2-FCV), ML | 0.89 (AUC) | 0.72-0.82/ 0.72-0.8 | 0.77-0,87/ 0.67-0.77 |
| **Pramparo et al. 2015^16^** | ASD vs HC | Cohort | 1.8, 0 | 215 ^a^ | 762: Genetic | I (10-FCV and random split), ML | 0.87 (AUC) | 0.72-0.82/ 0.72-0.8 | 0.77-0,87/ 0.67-0.77 |

^a^ In the case of studies performing an external validation, the development and external validation data are detailed if available; ^b^ Assessed with drug use survey ^c^ According to the internet addiction test^17^

ADHD: Attention Deficit and Hyperactivity Disorder; ARMS: At-risk mental state; ASP: Affective Spectrum Psychosis; ASD: Autism spectrum disorders; BD: Bipolar disorder; BPSD: Bipolar spectrum disorder; CHR-P: clinical high risk of psychosis; CV: cross-validation; Dev: Development database; Ext: External validation database; GAD: Generalised anxiety disorder; HC: Healthy controls; Int: Internal validation database; LLSO-CV: Leave Single Site Out cross-validation; ML: machine learning; MD: major depression; PD: Panic disorder; PTSD: Post-traumatic stress disorder; S: statistical; SCZ: schizophrenia; SSP: Schizophrenia Spectrum Psychoses; 2-FCV: 2-fold cross-validation 10-FCV: 10-fold cross-validation.

**eTable 4. Characteristics of prognostic risk estimation models**

| **Author** | **Outcome (risk of)** | **Data source** | **Mean age in years (SD or range),**  **% female** | **Sample size:**  **total** | **Predictors:**  **n: categories** | **Validation type (Dev/ Ext)^a^, Main analysis** | **Performance (measure)**^a^ | **Se; Sp** | **PPV; PNV** |
| --- | --- | --- | --- | --- | --- | --- | --- | --- | --- |
| **Bedi et al. 2015^18^** | Psychosis onset in CHR-P | Cohort | 21.4 (3.6), 32.3 | 34 | N.a: Cognitive | I (LSSO), ML | 1.0 (accuracy) | 1.0/1.0 | 1.0/1.0 |
| **Cannon et al. 2016^19^** | Psychosis onset in CHR-P | Cohort | 18.5 (4.3), 42.3 | 596 | 6: Sociodemographic, clinical, cognitive | I (bootstrap), S | 0.71 (C-index) | 0.48/0.84 | 0.33/0.9 |
| **Carrión et al. 2016^20^** |  | Cohort | 16.6 (3.3), 41.5 | 176 |  | E (geographical)  (n.a/ 176), S | 0.79 (AUC, Ext) | 0.5/0.82 | 0.17/0.96 |
| **Ciarleglio et al. 2019^21^** | Psychosis onset in CHR-P | Cohort | 20.08 (3.82), 27 | 199 | 17: Sociodemographic, clinical | I (5-FCV), ML | 0.73 (C-index) | 0.75/0.79 | 0.62/0.87 |
| **Malda et al. 2019^22^** | Psychosis onset in CHR-P | Registry data | 15.3 (1.3)- 25.3 (6.3), 26.6-68.2 | 1676 | 6: Sociodemographic, clinical | I (IECV), S | 0.66 (AUC) | N.a. | N.a. |
| **Perkins et al. 2015^23^** | Psychosis onset in CHR-P | Cohort | 18.4 (4.5), 41 | 888 | 4: Clinical | E (temporal) (296/ 592), S | 0.74 (AUC, Dev);  0.71 (AUC, Ext) | N.a. | N.a. |
| **Ramyead et al. 2016^24^** | Psychosis onset in CHR-P | Cohort | 26.3 (7.5), 37.7 | 53 | 21: EEG | I (nested 10-CV with 10 repetitions), ML | 0.78 (AUC) | 0,58/0,83 | N.a. |
| **Corcoran et al. 2018^25^** | Psychosis onset in CHR-P | Cohort | 18.0 (4.2), 40 | 130 | 4: Cognitive | E (LLSO-CV)  (93/ 37), ML | 0.87 (AUC, Dev);  0.72 (AUC, Ext) | N.a. | N.a. |
| **Koutsouleris et al. 2012^26^** | Psychosis onset in CHR-P | Cohort | 24.1 (5.7), 40.7 | 59 | N.a: Neuroimaging | I (RN-CV), ML | 0.67-0.92 (accuracy)^b^ | 0.8-0.95/ 0.5-1.0 | 0.83-0.92/ 0.71-1.0 |
| **Koutsouleris et al. 2012^27^** | Psychosis onset in CHR-P | Case-control | 25.2 (4.9), 35.9 | 78 | 12: Cognitive | I (RN-CV), ML | 0.88 (accuracy) | 0.8/0.96 | 0.89/0.92 |
| **Zarogianni et al. 2017^28^** | Psychosis onset in CHR-P | Cohort | 20.05 (2.6), 35.3 | 34 | N.a: Neuroimaging | I (nested LLSO-CV), ML | 0.91 (accuracy) | 1.0/0.82 | 0.63/1.0 |
| **Chung et al. 2019^29^** | Psychosis onset in CHR-P | Cohort | n.a, Unclear | 476 | 6: Sociodemographic, clinical, cognitive, neuroimaging | I (bootstrap), S | 0.7 (C-index) | N.a. | N.a. |
| **Fusar-Poli et al. 2016^30^** | Pretest risk of psychosis | Registry data | 23.1 (5.4), 44 | 710 | 3: Sociodemographic, service use | E (geographical)  (321/ 389), ML | 0.66 (C-index, Dev); 0.65 (C-index, Ext) | N.a. | N.a. |
| **Fusar-Poli et al. 2017^31^** | Transdiagnostic psychosis onset in secondary mental healthcare patients | Registry data | 33.0 (18.6), 49.1 | 91199 | 5: Sociodemographic, clinical | E (geographical) (33820/ 54716, S | 0.8 (C-index, Dev); 0.79 (C-index, Ext) | N.a. | N.a. |
| **Fusar-Poli et al. 2019^32^**^c^ |  | Registry data | 33.0 (18.6), 49.1 | 91199 |  | E (geographical) (33820/ 54716, S | 0.81 (C-index, Dev); 0.8 (C-index, Ext) | N.a. | N.a. |
| **Fusar-Poli et al 2019^33^** |  | Registry data | 36.3 (18.2), 49.7 | 47522 |  | E (geographical) (13702), S | 0.73 (C-index, Ext) | N.a. | N.a. |
| **Koutsouleris et al. 2009^34^** | Psychosis onset in CHR-P | Case-Control | 25.1 (5.8), 37.77 | 90 | 21: Neuroimaging | I (5-FCV), S | 0.82 (accuracy) | 0.76-0.9/ 0.8-0.92 | 0.79-0.84 / 0.77-0.96 |
| **De Wit et al. 2017^35^** | Functioning and symptoms in CHR-P | Case-control | 15.6 (2.32), 34.1 | 126 | 3: Clinical, neuroimaging, | I (LLSO), ML | 0.75 (AUC) | 0,69/0,94 | N.a. |
| **Koutsouleris et al. 2018^36^** | Disability in CHR-P | Case-control | 24.0 (5.1), 50.0 | 116 | N.a: Clinical, neuroimaging | I (nested LLSO-CV), ML | 0.83 (accuracy) | 0.83/0.82 | 0.86/0.79 |
| **Addington et al. 2010^37^** | Hospital admission in FEP | Cohort | 26, 34.5 | 606 | 12: Sociodemographic, clinical, service use | I (bootstrapping)  (297/ 309), S | 0.67-0.72 (C-index) | N.a. | N.a. |
| **Leighton et al. 2019^38^** | Employment, education or training status in FEP | Cohort | 24.9 (6.3), 33 | 162 | 5: Sociodemographic, clinical | E (temporal) (83/ 79), ML | 0.88 (AUC, Ext) | 0.82/0.88 | 0.82/0.88 |
| **Fond et al. 2019^39^** | Psychotic relapse in SCZ | Cohort | 32.6, 24 | 549 | N.a: Sociodemographic, clinical, cognitive, service use, physical health | I (5-FCV), ML | 0.64 (accuracy). | 0.71/0.45 | N.a. |
| **Austin et al. 2012^40^** | Mortality in SCZ | Registry data | 47, 45.7 | 94466 | 16: Sociodemographic, physical health | I (50- subsamples), S | 0.84 (AUC) | N.a. | N.a. |
| **Koutsouleris et al. 2018^36^** | Disability in ROD | Case-control | 26.1 (6.1), 54.2 | 120 | N.a: Clinical, neuroimaging | I (nested LLSO-CV), ML | 0.7 (accuracy) | 0.77/0.64 | 0.7/0.63 |
| **Bellon et al. 2011^41^** | MD onset in GP | Cohort | 49.2, 66 | 11891 | 12: Sociodemographic, clinical, physical health | E (geographical)  (4574/ 7317), S | 0.82 (C-index, Dev); 0.83 (C-index, Ext) | 0.73/0.73 | N.a. |
| **King et al. 2008^42^** | MD onset in GP | Cohort | 48.9, 65.9 | 6948 | 10: Sociodemographic, clinical, physical health | E (geographical)  (5216/1732), S | 0.79 (C-index, Dev); 0.71 (C-index, Ext) | 0.32-0.53/ 0.8-0.9 | N.a. |
| **Nigatu et al. 2016^43^** |  | Cohort | 43.8 (15.2), 56.1 | 29621 |  | E (geographical) S | 0.71 (AUC, Ext) | 0.26-0.58/ 0.72-0.91 | 0.37/ 0.92 |
| **Maarsingh et al. 2018^44^** | Persistence of MD | Cohort | 64.7, 67 | 467 | 7: Sociodemographic, clinical | E (geographical)  (270/ 197), S | 0.75 (AUC, Dev);  0.7 (AUC, Ext) | 0.93/0.31 | 0.48/0.86 |
| **Dinga et al. 2018^45^** | Persistence of MD | Cohort | 42.0 (12.2), 65 | 804 | 78: Sociodemographic, clinical, physical health | I (10-FCV), ML | 0.69 (AUC) | 0.47/N.a. | N.a. |
| **Rubenstein et al. 2007^46^** | Persistence of MD | Cohort | 44.1 (13.8), 71 | 1792 | 6: Sociodemographic, clinical, physical health | I (SS-CV), S | None | N.a. | N.a. |
| **Wang et al. 2014^47^** | Recurrence of MD | Cohort | 45.4 (0.3), 76.3 | 2711 | 19: Sociodemographic, clinical, physical health | E (geographical)  (1518/ 1195), ML | 0.75 (C-index, Dev); 0.72 (C-index, Ext) | N.a. | N.a. |
| **Klein et al. 2018^48^** | Recurrence of MD | Clinical trial | 46.8 (10.6), 74.5 | 444 | 4: Clinical | E (geographical)  (235/ 209), S | 0.56 (C-index, Dev); 0.59 (C-index, Ext) | 0.16-0.52/ 0.69-0.95 | 0.59-0.72/ 0.57-0.63 |
| **Hafeman et al. 2017**^49^ | BPSD onset in youth at family risk | Cohort | 14.2 (4.5), 49 | 412 | 7: Sociodemographic, clinical | I (bootstrap), S | 0.76 (AUC) | 0.21-0.82/0.49-0.95 | 0.15-0.32/ n.a. |
| **Bauer et al. 2019^50^** | Cognitive impairments in BD | Case-control | 11.9 (3.5), 45.8 | 227 | 17: Sociodemographic, cognitive | I (K-FCV), ML | 0.79 (AUC) | N.a. | N.a. |
| **Ryu et al. 2018^51^** | Suicide ideation in GP | Cohort | 49.8 (17), 57.6 | 11628 | 15: Sociodemographic, clinical, physical health | I (SS and 10-FCV), ML | 0.85 (AUC) | 0,77/0,79 | 0.79/0.78 |
| **Liu et al. 2016^52^** | Suicidal ideation in GP | Cohort | 44.51, 71.9 | 3035 | 6: Sociodemographic, clinical | E (geographical) (1741/ 1294), S | 0.83 (C- index, Dev); 0.81 (C- index, Ext) | 0,75/0.77 | 0.77/N.a. |
| **Simon et al. 2018^53^** | Suicide attempts after outpatient visits | Registry data | 46 (<13), 62 | 2960929 | 59: Sociodemographic, clinical, service use, physical health | I (CV), S | 0.86 (AUC) | 0.17-0.92/ 0.5-0.99; | 0.01-0.1/ 0.99 - 1.0 |
| **Walsh et al. 2018^54^** | Suicide attempts in adolescents | Cohort | 16.0 (3.0), 49 | 33610 | 10: Sociodemographic, clinical, physical health | I (bootstrap), ML | 0.83-0.94 (AUC)^d^ | N.a. | N.a. |
| **Tran et al. 2014^55^** | Suicidal behaviour risk | Cohort | 41.2, 50.7 | 7399 | N.a: Sociodemographic, clinical | I (10-FCV), S | 0.79 (AUC) | 0.7/0.72 | N.a. |
| **Kessler et al. 2015^56^** | Committed suicide in soldiers | Registry data | n.a, Unclear | 40820 | 73: Sociodemographic, clinical | I (10-FCV), ML | 0.89 (AUC) | N.a. | N.a. |
| **Russo et al. 2013^57^** | PTSD onset in post-hospitalized | Cohort | 38.2 (13.7), 32.6 | 878 | 10: Sociodemographic, clinical, service use | I (RSV), S | 0.72 (AUC) | 0.71/0.66 | 0.58/0.78 |
| **Papini et al. 2018^58^** | PTSD onset after hospitalization | Cohort | 46.7 (17.3), 36 | 271 | 41: Sociodemographic, clinical, physical health | I (10- RN 5-FCV), ML | 0.85 (AUC) | 0,69/0,83 | 0,65/0,86 |
| **Rosellini et al. 2018^59^** | PTSD onset after a natural disaster | Cohort | 50.3, 68.5 | 23907 | 67: Sociodemographic, clinical, physical health | I (10-FCV), ML | 0.79 (AUC) | 0.85-0.97/ N.a. | N.a./0.89-0.91 |
| **Galatzer-Levy et al. 2017^60^** | PTSD remission | Cohort | 31.0 (12.0), 42.4 | 152 | N.a: Sociodemographic, clinical, physical health | I (10-FCV), ML | 0.82 (AUC) | 0.7/0.75 | N.a. |
| **Karstoft et al. 2015^61^** | PTSD remission | Cohort | n.a (18-70), n.a, | 957 | 13: Sociodemographic, clinical, physical health | I (10-FCV), ML | 0.75 (AUC) | N.a. | N.a. |
| **Galatzer-Levy et al. 2014^62^** | PTSD remission | Cohort | 36.3 (12.0), 48.9 | 957 | 16: Clinical, service use | I (10-FCV), ML | 0.77 (AUC) | N.a. | N.a. |
| **Karstoft et al. 2015^63^** | PTSD features in soldiers | Cohort | 26.2, 0,05 | 561 | 9: Clinical | I (10-FCV), ML | 0.88 (AUC) | N.a. | N.a. |
| **King et al. 2011^64^** | GAD and PD onset in GP | Cohort | n.a. (18-75), 67.2 | 10045 | 9: Sociodemographic, clinical, physical health | E (geographical) (4905/ 5140), S | 0.75 (C-index, Dev); 0.71-0.81 (C- index, Ext) | 0.27-0.68/0.8-0.9 | N.a. |
| **Nigatu et al. 2019^65^** |  | Cohort | N.a (18-75), 55.3 | 24626 | 9: Sociodemographic, clinical, physical health | E (geographical), S | 0.62 (AUC, Ext) | 0.19-0.47/ 0.71-0.9 | 0.50/0.95 |
| **Liu et al. 2015^66^** | Recurrence of PD | Cohort | 44.8, 71.7 | 1681 | 11: Sociodemographic, clinical, physical health | E (geographical) (949/ 732), S | 0.79 (C-index, Dev); 0.73 (C-index, Ext) | 0.15-0.49/ 0.87-0.98 | 0.46-0.67/ 0.84-0.89 |
| **Ngo et al. 2019^67^** | Harmful drinking events in students^e^ | Cohort | n.a (16-49), 56 | 179165 | 11: Sociodemographic, clinical | I (bootstrapping), S | 0.86 (C-index) | 0.78/ 0.77**^f^** | 0.09/ 0.99**^f^** |
| **Afzali et al. 2019^68^** | Frequency of alcohol use in adolescents | Cohort | 12.8 (49.2), 49.2 | 6016 | 11: Sociodemographic, clinical, cognitive | E (geographical)  (3826/ 2190), ML | 0.87 (AUC, Dev);  0.86 (AUC, Ext) | N.a. | 0.85./0,79 |
| **Gueorguieva et al. 2014^69^** | Abstinence from heavy drinking | Clinical trial | n.a, n.a | 1646 | 4: Sociodemographic, clinical | E (geographical) (1220/ 426), S | 0.69 (AUC, Dev);  0.61 (AUC, Ext) | N.a. | N.a. |
| **Gueorguieva et al. 2015^70^** | Abstinence from heavy drinking | Clinical Trial | n.a, n.a | 1150 | 2: Clinical | I (RSV), S | 0.7-0.74 (AUC) | N.a. | N.a. |
| **Hickey et al. 2009^71^** | Acquisitive offending ^g^ in SCZ and DD. | Registry data | 31.7 (9.6), 10 | 1344 | 6: Clinical | I (RSV), S | 0.88 (AUC) | N.a. | N.a. |
| **Hotzy et al. 2018^72^** | Compulsory admission | Cohort | n.a, 48 | 393 | 18: Sociodemographic, clinical, service use | I (5-FCV), ML | 0.82 (AUC) | 0.71/ 0.79 | N.a. |
| **Muñoz et al. 2019^73^** | Medication-induced altered mental status in hospitalised | Cohort | 53.7 (17.9), 54.5 | 66875 | 16: Sociodemographic, clinical, service use. physical health | I (bootstrap), S | 0.86 (C-index) | N.a. | N.q. |
| **Fernandez et al. 2018**^74^ | CMD onset in a working population^h^ | Cohort | 41.4, 47.4 | 6189 | 8-13: Sociodemographic, clinical, physical health | I (RSV), S | 0.66-073 (C-index) | 0.62-0.64/ 0.75-0.78 | 0.26-0.28 |
| **Barker et al. 2018^75^** | Hospital readmission | Registry data | n.a, 50.7 | 65789 | 13: Sociodemographic, clinical, service use | I (RSV), S | 0.65 (C-index) | N.a. | N.a. |
| **Fazel et al. 2017^76^** | Violence offending^i^ in SMD | Registry data | 44, 51 | 75158 | 16: Sociodemographic, clinical, service use | E (geographical), S | 0.89 (C-index, Ext) | 0.62/0.94 | 0.11/0.99 |

^a^ In the case of studies performing an external validation, the development and external validation data are detailed if available; ^b^ Depending on comparison group: 0.92 HCs vs converters; 0.67 HCs vs non-converters; 0.84 converters vs non-converters; ^c^ Refined model with non-linear age effect; ^d^ Depending on comparison group: 0.83 other self-injury- 0.94 general hospital controls; ^e^ Alcohol intoxication associated with ED visits or alcohol-related incidents reported to authorities within 1 year following the annual (index) enrolment; ^f^ Performance to predict top 25%; ^g^ Defined as a conviction for any offence of theft, stealing, burglary, or fraud; ^h^ Two different models depending on sex; ^i^ Occurrence of any violent offending.

AG: Agoraphobia; ARMS: at-risk mental state; AUROC: area under the receiver operating curve; BPSD: Bipolar spectrum disorder; CHR-P: clinical high risk for psychosis; CMD: common mental disorder; DD: delusional disorder; Dev: development database; ED: emergency department; EXT: external validation database; FEP: first episode psychosis; GAD: generalized anxiety disorder; GGT: gamma-glutamyl transferase level; GP: general population; ICD: International Classification of Diseases; IECV: internal-external cross validation; Int: internal validation database; IQ: intelligence quotient; k-FCV: k-fold cross-validation; ML: machine learning; LLSO: Leave Single Site Out; LLSO-CV: Leave Single Site Out cross validation; MD: major depression; MDE: major depressive episode; PD: panic disorder; PTSD: posttraumatic stress disorder; RN-CV: repeated nested cross-validation; ROD: recent-onset depression RSV: random split validation; S: statistical; SCZ: schizophrenia; SMD: severe mental disorder; SS: split-sample; SS-CV: split-sample cross-validation; 5-FCV: 5-fold cross-validation; 10-FCV: 10-fold cross-validation.

**eTable 5: Characteristics of predictive risk estimation models**

| **Author** | **Outcome** | **Data source** | **Mean age in years**  **(SD or range), % female** | **Sample size:**  **total** | **N: Type of predictors** | **Validation type**  **(Dev / Ext)^a^, Main analysis** | **Performance (measure) (Dev; Ext)** ^a^ | **Se; Sp** | **PPV; PNV** |
| --- | --- | --- | --- | --- | --- | --- | --- | --- | --- |
| **Chekroud et al. 2016^77^** | Remission in MD | Clinical trial | n.a (18-75), Unclear | 2374 | 25: Sociodemographic, clinical, physical health | E (independent trial)  (1949/ 425), ML | 0.7 (AUC, Dev); n.a | 0.39-0.56/ 0.63-0.71 | 0.54-0.65/ 0.5-0.6 |
| **Furukawa et al. 2019^78^** | Remission in MD | Clinical trial | 42.5, 53.3 | 2011 | 6: Sociodemographic, clinical, | E (temporal) (1009/ 1002), S | 0.85 (AUC, Dev); 0.82 (AUC, Ext)^a^ | 0.17-0.79/ 0.55 -0.97 | 0.51-0.83/ 0.66-0.84 |
| **Maciukiewicz et al. 2018^79^** | AD response in MD | Clinical trial | 46.7 (12.4), 67.9 | 186 | N.a: Genetic | I (nested 5-FCV), ML | 0.66 (accuracy) | 0.7/0.61 | N.a. |
| **Serretti et al. 2007^80^** | AD response in MD | Clinical trial | 50.97 (13.4), 76.7 | 116 | 15: Sociodemographic, clinical, physical health | I (split-sample and LLSO-CV), S | 0.77 (AUC) | N.a. | N.a. |
| **Perlis et al. 2013^81^** | TRD | Clinical trial | 41.32 (13.04), 61.6 | 2555 | 14: Sociodemographic, clinical | E (geographical) (2094/ 461), ML | 0.71 (AUC Dev); 0.72 (AUC, Int) | 0.26/0.91 | 0.61/0.68 |
| **Kautzky et al. 2019^82^** | TRD | Cohort | 52.63, 64.4 | 916 | 8: Clinical, service use | E (geographical) (602/ 314), S | 0.87 (accuracy, Ext) | 0.95/0.78 | 0.82/ 0.93 |
| **Kautzky et al. 2018^83^** | TRD | Cohort | 52.8 (14.3), 64.91 | 552 | 47: Sociodemographic, clinical, service use, physical health | I (10-FCV), ML | 0.75 (accuracy) | 0.82/0.63 | 0.8/0.68 |
| **Koutsouleris et al. 2016^84^** | Functioning in FEP | Clinical trial | 26.1 (5.6), 40 | 489 | 10: Sociodemographic, clinical | I (LLSO-CV), ML | 0.75 4-weeks; 0.74 52-weeks (accuracy) | 0.74;0.76/ n.a. | 0.86/ 0.59 |
| **Zandvakili et al. 2019^85^** | Response to TMS in comorbid PTSD and MD | Clinical trial | 51.6 (10.3), 40 | 29 | 5 MDD; 8 PTSD: EEG | I (LLSO-CV and feature bagging), ML | 0.83 MDD; 0.71 PTSD (AUC) | 1.0/ 0.46 MDD; 0.94/ 0.5 PTSD | N.a. |
| **Koutsouleris et al. 2018^86^** | Response to TMS in SCZ | Clinical trial | 34.8 (9.7), 17.4 | 92 | N.a: Neuroimaging | I (Nested LLSO-CV), ML | 0.92 (AUC) | 0.79/0.9 | 0.91/0.79 |
| **Erguzel et al. 2015^87^** | Response to TMS in MD | Clinical trial | n.a, Unclear | 55 | N.a: Neuroimaging, EEG | I (10-FCV), ML | 0.89 (AUC) | 0.93/ 0.8 | N.a. |
| **Niles et al. 2017^88^** | Treatment dropout in CBT and ACT in ADS | Clinical trial | 34.1 (11), 50 | 208 | 4: Clinical, physical health | I (K-FCV), S | None | N.a. | N.a. |

^a^ Performance at week 3; ^b^ CV sample 0.87 (C-index).

ACT acceptance and commitment therapy; AD: antidepressant; ADS: anxiety disorders; ANN: artificial neural network; BD: bipolar disorder; BMI: body mass index; CBT: cognitive behavioral therapy; Dev: development database; EEG: Electroencephalography; Ext: External validation database; FEP: First episode psychosis; GAD: generalized anxiety disorder; GWAS: genome-wide association study; Int: Internal validation database; LLSO-CV: Leave Single Site Out cross validation; K-FCV: k-fold cross-validation; MD: major depression; ML: machine learning; PTSD: post-traumatic stress disorder; rTMS: repetitive transcranial magnetic stimulation; S: Statistical; SCZ: schizophrenia; TRD: treatment resistant depression; TMS: transcranial magnetic stimulation; 5-FCV: 5-fold cross-validation10-FCV: 10-fold cross-validation.

**eTable 6** Meta-regressions between accuracy and different moderating factors

| **Moderating factors** | **N studies** **(subgroups)** | **Meta-regression Coefficient** | **SE** | **Z value** | **P** | **95%CI (low; high)** |
| --- | --- | --- | --- | --- | --- | --- |
| (i) Type of validation |  |  |  |  |  |  |
| External vs Internal validation | 76 | -0.0092 | 0.020 | -0.36 | 0.71 | -0.059; 0.041 |
| (ii) Type of accuracy measure | | | | | | |
| Accuracy vs AUC | 61 (15;46) | 0.22 | 0.25 | 0.91 | 0.36 | -0.26; 0.71 |
| Accuracy vs c-index | 30 (15;15) | 0.044 | 0.27 | 0.16 | 0.87 | -0.48; 0.57 |
| AUC vs c-index | 61 (46;15) | -0.27 | 0.17 | -1.56 | 0.12 | -0.61;0.068 |
| (iii) Type of model | | | | | | |
| Diagnostic vs prognostic | 65 (14;51) | 0.84 | 0.21 | 3.98 | **<0.001** | 0.42;1.25 |
| Diagnostic vs predictive | 25 (14;11) | 0.87 | 0.28 | 3.06 | **0.002** | 0.31; 1.43 |
| Prognostic vs predictive | 62 (51;11) | -0.030 | 0.22 | -0.13 | 0.89 | -0.47;0.41 |
| (iv) Number of predictors | | | | | | |
| Number of predictors | 56 | 0.0030 | 0.005 | 0.65 | 0.52 | -0.006; 0.010 |
| Number of predictors by type of validation | 56 | 0.0024 | 0.005 | 0.49 | 0.62 | -0.007;0.012 |
| Number of predictors by type of accuracy measure | 56 | 0.0037 | 0.005 | 0.75 | 0.45 | -0.88;0.52 |
| (v) Type of predictor | | | | | | |
| Clinical or service use or sociodemographic vs biomarkers | 67 (49;18) | -0.12 | 0.15 | -0.82 | 0.43 | -0.41; 0.18 |
| Clinical or service use or sociodemographic vs combination | 58 (49;9) | -0.080 | 0.20 | -0.40 | 0.69 | -0.47; 0.31 |
| Biomarkers vs combination | 27 (18;9) | 0.19 | 0.31 | 0.61 | 0.54 | -0.42; 0.81 |
| Type of predictor by type of validation | 76 (49;18;9) | 0.29 | 0.16 | 1.78 | 0.075 | -0.60; 0.029 |
| Type of predictor by type of accuracy measure | 76 (49;18;9) | 0.22 | 0.26 | -0.85 | 0.40 | -0.29; 0.73 |
| (vi) Modality of predictors | | | | | | |
| Unimodal vs Multimodal | 76 (25; 71) | 0.29 | 0.14 | 2.14 | **0.030** | 0.02; 0.56 |
| Modality of predictors by type of validation | 76 (25; 71) | -0.37 | 0.19 | -1.95 | 0.051 | -0.74; 0.002 |
| Modality of predictors by type of accuracy measure | 76 (25; 71) | -0.34 | 0.19 | -1.83 | 0.067 | -0.71; 0.024 |
| (vii) Type of analysis | | | | | | |
| Machine learning vs statistical modelling | 76 (46;30) | -0.12 | 0.15 | -0.82 | 0.41 | -0.41; 0.17 |
| Type of analysis by type of validation | 76 (46;30) | -0.27 | 0.16 | -1.66 | 0.097 | -0.59; 0.049 |
| Type of analysis by type of accuracy measure | 76 (46;30) | -0.076 | 0.17 | -0.45 | 0.65 | -0.41; 0.26 |

**eTable 7: PROBAST Quality assessment results***

|  | **Participants**  **Risk of bias** | **Predictors**  **Risk of bias** | **Outcomes**  **Risk of bias** | **Analysis**  **Risk of bias** | **Overall**  **Risk of bias** |
| --- | --- | --- | --- | --- | --- |
| **Kalmady et al. 2019^1^** | Low | Low | High | High | High |
| **Rozycki et al. 2018^2^** | Low | Low | High | High | High |
| **Cooper et al. 2019^3^** | Low | Low | High | High | High |
| **Viviano et al. 2018^4^** | Low | High | High | High | High |
| **Jauhar et al. 2018^5^** | Low | Low | High | High | High |
| **Redlich et al. 2014^6^** | High | High | High | High | High |
| **Lueken et al. 2015^7^** | Low | High | High | High | High |
| **Parker et al. 2015^8^** | Low | Low | High | High | High |
| **Ding et al. 2017^9^** | Low | Low | High | High | High |
| **Ioannidis et al. 2016^11^** | Low | High | High | High | High |
| **Harrington et al. 2019^12^** | Low | Low | High | Low | High |
| **James et al. 2015^13^** | Low | Low | High | High | High |
| **Duda et al. 2016^14^** | Low | High | High | Low | High |
| **Duda et al. 2017^15^** | Low | Low | High | High | High |
| **Bedi et al. 2015^18^** | Low | High | High | High | High |
| **Cannon et al. 2016^19^** | Low | High | High | High | High |
| **Carrión et al. 2016^20^** | Low | High | High | High | High |
| **Ciarleglio et al. 2019^21^** | Low | High | High | High | High |
| **Malda et al. 2019^22^** | Low | High | High | High | High |
| **Perkins et al. 2015^23^** | Low | Low | High | High | High |
| **Ramyead et al. 2016^24^** | Low | Low | High | High | High |
| **Corcoran et al. 2018^25^** | Low | Low | High | High | High |
| **Koutsouleris et al. 2012^26^** | Low | High | High | High | High |
| **Koutsouleris et al. 2012^27^** | Low | High | High | High | High |
| **Zarogianni et al. 2017^28^** | Low | High | High | High | High |
| **Chung et al. 2019^29^** | Low | High | High | High | High |
| **Fusar-Poli et al. 2016^30^** | Low | High | High | Low | High |
| **Fusar-Poli et al. 2017^31^** | Low | High | High | Low | High |
| **Fusar-Poli et al. 2019^32^** | Low | High | High | Low | High |
| **Fusar-Poli et al. 2019^33^** | Low | High | High | Low | High |
| **Koutsouleris et al. 2009^34^** | Low | High | High | High | High |
| **De Wit et al. 2017^35^** | Low | Low | Low | High | High |
| **Addington et al. 2010^37^** | Low | High | High | High | High |
| **Leighton et al. 2019^38^** | Low | High | High | High | High |
| **Fond et al. 2019^39^** | Low | Low | High | High | High |
| **Austin et al. 2012^40^** | Low | Low | High | High | High |
| **Koutsouleris et al. 2018^86^** | Low | High | High | High | High |
| **Bellon et al. 2011^41^** | Low | High | High | High | High |
| **King et al. 2008^42^** | Low | High | High | High | High |
| **Nigatu et al. 2016^43^** | Low | High | High | High | High |
| **Maarsingh et al. 2018^44^** | Low | Low | Low | Low | Low |
| **Dinga et al. 2018^45^** | Low | High | High | High | High |
| **Rubenstein et al. 2007^46^** | Low | High | High | High | High |
| **Wang et al. 2014^47^** | Low | Low | High | High | High |
| **Klein et al. 2018^48^** | Low | High | High | Low | High |
| **Hafeman et al. 2017^49^** | Low | Low | High | Low | High |
| **Bauer et al. 2019^50^** | Low | High | High | High | High |
| **Ryu et al. 2018^51^** | Low | High | High | High | High |
| **Liu et al. 2016^52^** | Low | High | High | High | High |
| **Simon et al. 2018^53^** | Low | High | High | High | High |
| **Walsh et al. 2018^54^** | Low | High | High | High | High |
| **Tran et al. 2014^55^** | Low | High | High | High | High |
| **Kessler et al. 2015^56^** | Low | High | High | High | High |
| **Russo et al. 2013^57^** | Low | High | High | High | High |
| **Papini et al. 2018^58^** | Low | Low | High | High | High |
| **Rosellini et al. 2018^59^** | Low | High | Low | High | High |
| **Galatzer-Levy et al. 2017^60^** | Low | High | High | High | High |
| **Karstoft et al. 2015^61^** | Low | Low | Low | Low | Low |
| **Galatzer-Levy et al. 2014^62^** | Low | High | High | High | High |
| **Karstoft et al. 2015^63^** | Low | High | High | Low | High |
| **King et al. 2011^64^** | Low | High | High | High | High |
| **Nigatu et al. 2019^65^** | Low | High | High | High | High |
| **Liu et al. 2015^66^** | Low | High | High | High | High |
| **Ngo et al. 2019^67^** | Low | High | High | High | High |
| **Afzali et al. 2019^68^** | Low | High | High | Low | High |
| **Gueorguieva et al. 2014^69^** | Low | High | High | High | High |
| **Gueorguieva et al. 2015^70^** | Low | High | High | High | High |
| **Pramparo et al. 2015^16^** | Low | Low | Low | Low | Low |
| **Hickey et al. 2009^71^** | Low | High | High | High | High |
| **Hotzy et al. 2018^72^** | Low | Low | High | High | High |
| **Muñoz et al. 2019^73^** | Low | High | High | Low | High |
| **Fernandez et al. 2018^74^** | Low | High | High | High | High |
| **Barker et al. 2018^75^** | Low | High | High | High | High |
| **Fazel et al. 2017^76^** | Low | Low | High | High | High |
| **Chekroud et al. 2016^77^** | Low | High | High | High | High |
| **Furukawa et al. 2019^78^** | Low | High | High | High | High |
| **Maciukiewicz et al. 2018^79^** | Low | High | High | High | High |
| **Serretti et al. 2007^80^** | Low | High | Low | High | High |
| **Perlis et al. 2013^81^** | Low | Low | High | High | High |
| **Kautzky et al. 2019^82^** | Low | Low | Low | Low | Low |
| **Kautzky et al. 2018^83^** | Low | Low | Low | Low | Low |
| **Koutsouleris et al. 2016^84^** | Low | High | High | High | High |
| **Zandvakili et al. 2019^85^** | Low | High | High | High | High |
| **Erguzel et al. 2015^87^** | Low | High | High | High | High |
| **Niles et al. 2017^88^** | Low | Low | High | High | High |

*****To be considered at low risk of bias all questions should be answered as appropriate^89, 90^

**eMethods 1: Search terms used in the literature search**

Web of Science, Cochrane Central Register of Reviews, Ovid/ PsycINFO database and OpenGrey database were searched, from inception until 21th July 2019, in English. We used the following search terms: “risk prediction” OR “predictive model” OR “prognostic model” OR “individualised risk” OR “random forest” OR “neural network” OR “classification tree” OR “regression tree” OR “estimation tree” OR “elastic net” OR “deep learning” OR “estimation algorithm” OR “machine learning” OR “vector machine” OR “boosting” OR “lasso” OR “ridge” OR “regression” OR “logistic regression” OR “cox regression” and filtering for the Web of Science categories “psychiatry”, “psychology”, “psychology experimental” or “psychology clinical”. The records identified during this step were recorded. We then repeated the search using the same keyword plus the function AND (“validat*” OR “implement*”) to identify those studies that had been fully validated or implemented, in line with the inclusion criteria.

Literature search, screening for inclusion and exclusion criteria and data extraction were conducted by at least two independent researchers for each article (GSP, JV, JI, AC). Consensus was reached through discussion consulting a senior researcher (PFP).

50698 records were identified, and their abstracts were then screened to identify studies that were reporting prediction models that were developed (even without any type of validation). 49665 records were excluded at this stage, leaving 1033 records that were inspected reading the full article. Of these, 584 risk estimation models that were developed were finally identified.

**eMethods 2: Exclusion of models according to internal or external validation procedures**

Internal validation, as defined by Royston and Altman, “means reusing parts or all of the dataset on which a model was developed to assess the likely overfit and to correct for resulting ‘optimism’ in the performance of the model”^91^.

This criterion excludes pseudo-cross-validation^92^, such as studies that 'double-dip' by selecting predictors and optimal cut-offs using the whole sample instead of the development dataset. Pseudo-cross-validation also encompasses studies that (i) perform internal validation but then report the apparent model's performance instead of the cross-validated predictive performance, (ii) report size and significance of regression coefficients without cross-validated performance measures, or (iii) re-estimate regression coefficients in the test dataset instead of applying the internally validated model as it was.

External validation, again, as defined by Royston and Altman, refers to the assessment of an already developed model´s performance when applied to an independent dataset^91^. Accordingly, this does not include (i) studies that repeat the whole modelling process on new data with the aims of comparing variables selected in the model, comparing regression coefficients and/or assessing goodness of fit^91^, and (ii) studies that refit models on the independent dataset based on those predictors^91^ selected from the initial modelling process in the derivation dataset. Studies that removed some of the original predictors in the external validation analysis and studies that employed imputing techniques to measure the original predictors in the external validation analysis were considered as external validation studies.

**eMethods 3: Variables included in the study**

The following variables were included from the “Checklist for critical Appraisal and data extraction for systematic Reviews of prediction Modelling Studies” (CHARMS)^93^: Source of data (cohort, case control, clinical trial or registry data); Setting; Participant eligibility and recruitment method; Participant description; Treatments received; Study dates; Definition and method for measurement of outcome ; Type of analysis (machine learning, statistical learning) Type of outcome (single or multiple endpoints); Predictors part of outcome or not; Time of outcome occurrence or summary of duration of follow-up; Number of predictors (tested and final); Type of predictors; Selection of predictors; Sample size; Number of events (in development and validation group); Quantity of missing data; Handling of missing data; Model development; Modelling method; Methods for selection of predictors; Calibration measures; Discrimination measures and results; Classification measures (sensitivity, specificity, predictive values); Method used for testing model performance; Blinding of Predictors; Handling of Predictors; Satisfaction of Modelling assumptions satisfied or not; Shrinkage of predictor weighs or regression coefficients; Calibration validation; Type of validation (internal and external); Discrimination measures and results (derivation and external validation); Model adjustment (if applicable); Multivariable model (predictors included); Interpretation of presented models; Discussion of the results.

We expanded these variables and included the following as well: First author and year of publication; Study type (diagnostic, prognostic, predictive); Topic and mental disorder investigated; Mean age; Sex; Time points; Statistical analysis; Model presentation; Summary description of the risk estimation model; Consistency of outcome definition.

**eMethods 4: Quality assessment and risk of bias items (PROBAST v5/05/2019**^90, 94^**)**

PROBAST includes four steps: specify the systematic review question; classify the type of prediction model evaluation (e.g. internal or external validation; assess risk of bias and applicability of core domains (participants, predictors, outcome, and analysis) and overall judgement (low, high or unclear)^94^.

The review question is detailed in the introduction of this manuscript; the type of prediction model for each of the studies is detailed in the methods; risk of bias and applicability were assessed with the PROBAST questions (see below). We finally classified the models as “low risk/ high risk” for each of the domains. To be considered at low risk of bias all questions should be answered as appropriate (yes or probably yes)^89, 90^. An outcome is considered to be at high risk of bias when at least one of the questions in answered as not appropriate (no or probably no). The overall risk of bias is considered high risk when one or more domains is considered at high risk^89^.

Step 3 PROBAST Questions:

**Participants:**

- 1. Were appropriate data sources used, e.g. cohort, RCT or nested case-control study data?
  2. Were all inclusions and exclusions of participants appropriate?

**Predictors:**

- 1. Were predictors defined and assessed in a similar way for all participants?
  2. Were predictor assessments made without knowledge of outcome data?
  3. Are all predictors available at the time the model is intended to be used?

**Outcomes:**

- 1. Was the outcome determined appropriately?
  2. Was a pre-specified or standard outcome definition used?
  3. Were predictors excluded from the outcome definition?
  4. Was the outcome defined and determined in a similar way for all participants?
  5. Was the outcome determined without knowledge of predictor information?
  6. Was the time interval between predictor assessment and outcome determination appropriate?

**Analysis:**

- 1. Were there a reasonable number of participants with the outcome?
  2. 4.2 Were continuous and categorical predictors handled appropriately?
  3. Were all enrolled participants included in the analysis?
  4. Were participants with missing data handled appropriately?
  5. Was selection of predictors based on univariable analysis avoided?
  6. Were complexities in the data (e.g. censoring, competing risks, sampling of controls) accounted for appropriately?
  7. Were relevant model performance measures evaluated appropriately?
  8. Were model overfitting and optimism in model performance accounted for?
  9. Do predictors and their assigned weights in the final model correspond to the results from multivariable analysis?

**eMethods 5: Core descriptive variables reported in the summary tables**

Core descriptive variables detailed in the summary tables of the current systematic review included: author and year, outcome, data source, age, % female, sample size, broader type of predictors (clinical, sociodemographic, service use, neuroimaging, electroencephalography, magnetoencephalography, proteomic, genetic, cognitive), validation type (internal vs external and type), method (machine learning, statistical learning), risk of bias (see below), performance and measure, sensibility, specificity, positive predictive value and negative predictive value.

**eMethods 6: Categories and coding of machine learning and statistical learning**

We followed previously established criteria published in the British Medical Journal to classify the type of analysis as statistical learning or machine learning^95^:

-Statistical learning included cox hazard model, logistic regression model, linear regression model, negative binomial model, generalised linear model, weibull regression model, regularised regression model and other regression methods, either standard, penalised, boosted or bagged.

-Machine learning included classification trees, random forests, artificial neural networks, support vector machines, boosted tree methods, Bayes machine learning algorithms, K nearest neighbours algorithms, multivariate adaptive regression splines and genetic algorithms.

**eResults: Meta-regression analyses**

Type of validation (external vs internal validation: β=-0.009, p=0.71), type of accuracy measure (accuracy, n=15 vs AUC, n=46: β=0.22, p=0.36; accuracy, n=15 vs C-index, n=15: β=0.044, p=0.87; AUC, n=46 vs C-index, n=15: β=-0.269, p=0.118) (Table 1), number of predictors (β=0.003; p=0.52) (Table 1), type of predictors (clinical or service use or sociodemographic, n=49 vs biomarkers, n=18: β=-0.12, p=0.43; clinical or service use or sociodemographic, n=49 vs combination, n=9: β=-0.08, p=0.69; biomarkers, n=18 vs combination, n=9: β=0.19, p=0.54) (Table 1) and type of analysis (machine-learning methods, n=46 vs statistical modelling, n=30: β=-0.12, p=0.41) (Table 1).

Accuracy was higher in unimodal (n=25) vs multimodal (n=71) prediction models (β=0.29, p=0.03) (Table 1). Accuracy was higher in diagnostic (n=14) vs prognostic (n=51: β=0.84, p<0.001) models and in diagnostic (n=14) vs predictive (n=11: β=0.87, p=0.002) models (eTable 5).

There was no interaction between the number of predictors, type of predictors, the modality of predictors and type of analysis and type of validation or type of accuracy measure (all interactions p>0.05, eTable 5).

**eLimitations: Limitations of the study**

The main limitation of our study is the heterogeneity of the characteristics of prediction models developed in the included studies. For example, the type of the disorders studied, and the predictors used to develop the models vary considerably. Because of this, we did not attempt meta-analyses pooling categories of prediction models but presented only meta-regression analyses stratifying the models for general methodological features. The quality of the studies is also very heterogeneous, and the risk of bias results high in all the included studies: 65.9% of the studies are at high risk of bias in the predictors' domain, 90.9% in the outcomes domain and 81.8% in the analysis domain according to the PROBAST criteria. Another limitation is that only 30.3% of the prediction models that were included in this review were externally validated. Other methodology issues include the lack of handling of missing data in 51.1% of the studies and the fact that 46.6% of the studies did not present any details of their model, limiting their reproducibility. Furthermore, about 47.7% did not present calibration results. Although our meta-regression analyses mostly focused on accuracy, calibration remains equally relevant to improve clinical utility.

We did not systematically contact authors to retrieve the missing data. However, most studies were excluded because they did not meet the inclusion criteria for proper internal or external validation. As such it is unlikely that a study which was not providing individualised estimates would have additionally passed this stringent methodological filter.

Another limitation is that the amount of studies describing diagnostic and predictive risk estimation models is low compared to prognostic models and, as mentioned in the discussion, there were no studies describing the implementation of prediction models. Importantly, the 88 studies included in the current review may not be exhaustive of the whole prediction models that have been developed but never validated or of the whole psychiatric literature. The latter point is related to the fact that other individualised prediction models of interest for psychiatry may have been published under different Web of Knowledge categories (e.g. in the category “multidisciplinary sciences”)^96^. However, screening the whole Web of Knowledge literature is unfeasible and lead to the problem of spurious prediction models that would not directly apply to psychiatric knowledge. Finally, there might be a gap from when articles are published online and when they are uploaded in Web of Knowledge.

**eFigure 1. Risk of bias among the retrieved studies**

**REFERENCES**

**1.** Kalmady SV, Greiner R, Agrawal R, et al. Towards artificial intelligence in mental health by improving schizophrenia prediction with multiple brain parcellation ensemble-learning. *Npj Schizophrenia* Jan 2019;5.

**2.** Rozycki M, Satterthwaite TD, Koutsouleris N, et al. Multisite Machine Learning Analysis Provides a Robust Structural Imaging Signature of Schizophrenia Detectable Across Diverse Patient Populations and Within Individuals. *Schizophrenia Bulletin* Sep 2018;44(5):1035-1044.

**3.** Cooper JD, Han SYS, Tomasik J, Ozcan S, Rustogi N, van Beveren NJM, Leweke FM, Bahn S. Multimodel inference for biomarker development: an application to schizophrenia. *Translational Psychiatry* Feb 2019;9.

**4.** Viviano JD, Buchanan RW, Calarco N, et al. Resting-State Connectivity Biomarkers of Cognitive Performance and Social Function in Individuals With Schizophrenia Spectrum Disorder and Healthy Control Subjects. *Biological Psychiatry* Nov 2018;84(9):665-674.

**5.** Jauhar S, Krishnadas R, Nour MM, Cunningham-Owens D, Johnstone EC, Lawrie SM. Is there a symptomatic distinction between the affective psychoses and schizophrenia ? A machine learning approach. *Schizophrenia Research* Dec 2018;202:241-247.

**6.** Redlich R, Almeida JJ, Grotegerd D, et al. Brain morphometric biomarkers distinguishing unipolar and bipolar depression. A voxel-based morphometry-pattern classification approach. *JAMA Psychiatry* Nov 2014;71(11):1222-1230.

**7.** Lueken U, Straube B, Yang YB, et al. Separating depressive comorbidity from panic disorder: A combined functional magnetic resonance imaging and machine learning approach. *Journal of Affective Disorders* Sep 2015;184:182-192.

**8.** Parker G, McCraw S, Hadzi-Pavlovic D. The utility of a classificatory decision tree approach to assist clinical differentiation of melancholic and non-melancholic depression. *Journal of Affective Disorders* Jul 2015;180:148-153.

**9.** Ding XY, Yang YH, Stein EA, Ross TJ. Combining Multiple Resting-State fMRI Features during Classification: Optimized Frameworks and Their Application to Nicotine Addiction. *Frontiers in Human Neuroscience* Jul 2017;11.

**10.** Ahn WY, Ramesh D, Moeller FG, Vassileva J. Utility of Machine-Learning Approaches to Identify Behavioral Markers for Substance Use Disorders: Impulsivity Dimensions as Predictors of Current Cocaine Dependence. *Front Psychiatry* 2016;7:34.

**11.** Ioannidis K, Chamberlain SR, Treder MS, et al. Problematic internet use (PIU): Associations with the impulsive-compulsive spectrum. An application of machine learning in psychiatry. *Journal of Psychiatric Research* Dec 2016;83:94-102.

**12.** Harrington KM, Quaden R, Stein MB, et al. Validation of an Electronic Medical Record-Based Algorithm for Identifying Posttraumatic Stress Disorder in US Veterans. *Journal of Traumatic Stress* Apr 2019;32(2):226-237.

**13.** James LM, Belitskaya-Levy I, Lu Y, Wang H, Engdahl BE, Leuthold AC, Georgopoulos AP. Development and application of a diagnostic algorithm for posttraumatic stress disorder. *Psychiatry Research-Neuroimaging* Jan 2015;231(1):1-7.

**14.** Duda M, Ma R, Haber N, Wall DP. Use of machine learning for behavioral distinction of autism and ADHD. *Translational Psychiatry* Feb 2016;6.

**15.** Duda M, Haber N, Daniels J, Wall DP. Crowdsourced validation of a machine-learning classification system for autism and ADHD. *Translational Psychiatry* May 2017;7.

**16.** Pramparo T, Pierce K, Lombardo MV, et al. Prediction of Autism by Translation and Immune/Inflammation Coexpressed Genes in Toddlers From Pediatric Community Practices. *Jama Psychiatry* Apr 2015;72(4):386-394.

**17.** Moon SJ, Hwang JS, Kim JY, Shin AL, Bae SM, Kim JW. Psychometric Properties of the Internet Addiction Test: A Systematic Review and Meta-Analysis. *Cyberpsychol Behav Soc Netw* Aug 2018;21(8):473-484.

**18.** Bedi G, Carrillo F, Cecchi GA, et al. Automated analysis of free speech predicts psychosis onset in high-risk youths. *Npj Schizophrenia* 2015;1.

**19.** Cannon TD, Yu CH, Addington J, et al. An Individualized Risk Calculator for Research in Prodromal Psychosis. *American Journal of Psychiatry* Oct 2016;173(10):980-988.

**20.** Carrion RE, Cornblatt BA, Burton CZ, et al. Personalized Prediction of Psychosis: External Validation of the NAPLS-2 Psychosis Risk Calculator With the EDIPPP Project. *American Journal of Psychiatry* Oct 2016;173(10):989-996.

**21.** Ciarleglio AJ, Brucato G, Masucci MD, et al. A predictive model for conversion to psychosis in clinical high-risk patients. *Psychol Med* May 2019;49(7):1128-1137.

**22.** Malda A, Boonstra N, Barf H, et al. Individualized Prediction of Transition to Psychosis in 1,676 Individuals at Clinical High Risk: Development and Validation of a Multivariable Prediction Model Based on Individual Patient Data Meta-Analysis. *Frontiers in Psychiatry* May 2019;10.

**23.** Perkins DO, Jeffries CD, Cornblatt BA, et al. Severity of thought disorder predicts psychosis in persons at clinical high-risk. *Schizophr Res* Dec 2015;169(1-3):169-177.

**24.** Ramyead A, Studerus E, Kometer M, Uttinger M, Gschwandtner U, Fuhr P, Riecher-Rössler A. Prediction of psychosis using neural oscillations and machine learning in neuroleptic-naïve at-risk patients. *World J Biol Psychiatry* 06 2016;17(4):285-295.

**25.** Corcoran CM, Carrillo F, Fernandez-Slezak D, Bedi G, Klim C, Javitt DC, Bearden CE, Cecchi GA. Prediction of psychosis across protocols and risk cohorts using automated language analysis. *World Psychiatry* Feb 2018;17(1):67-75.

**26.** Koutsouleris N, Borgwardt S, Meisenzahl EM, Bottlender R, Moller HJ, Riecher-Rossler A. Disease Prediction in the At-Risk Mental State for Psychosis Using Neuroanatomical Biomarkers: Results From the FePsy Study. *Schizophrenia Bulletin* Nov 2012;38(6):1234-1246.

**27.** Koutsouleris N, Davatzikos C, Bottlender R, et al. Early recognition and disease prediction in the at-risk mental states for psychosis using neurocognitive pattern classification. *Schizophr Bull* Nov 2012;38(6):1200-1215.

**28.** Zarogianni E, Storkey AJ, Johnstone EC, Owens DGC, Lawrie SM. Improved individualized prediction of schizophrenia in subjects at familial high risk, based on neuroanatomical data, schizotypal and neurocognitive features. *Schizophrenia Research* Mar 2017;181:6-12.

**29.** Chung Y, Addington J, Bearden CE, et al. Adding a neuroanatomical biomarker to an individualized risk calculator for psychosis: A proof-of-concept study. *Schizophrenia Research* Jun 2019;208:41-43.

**30.** Fusar-Poli P, Rutigliano G, Stahl D, Schmidt A, Ramella-Cravaro V, Hitesh S, McGuire P. Deconstructing Pretest Risk Enrichment to Optimize Prediction of Psychosis in Individuals at Clinical High Risk. *Jama Psychiatry* Dec 2016;73(12):1260-1267.

**31.** Fusar-Poli P, Rutigliano G, Stahl D, Davies C, Bonoldi I, Reilly T, McGuire P. Development and Validation of a Clinically Based Risk Calculator for the Transdiagnostic Prediction of Psychosis. *Jama Psychiatry* May 2017;74(5):493-500.

**32.** Fusar-Poli P, Davies C, Rutigliano G, Stahl D, Bonoldi I, McGuire P. Transdiagnostic Individualized Clinically Based Risk Calculator for the Detection of Individuals at Risk and the Prediction of Psychosis: Model Refinement Including Nonlinear Effects of Age. *Frontiers in Psychiatry* May 2019;10.

**33.** Fusar-Poli P, Werbeloff N, Rutigliano G, Oliver D, Davies C, Stahl D, McGuire P, Osborn D. Transdiagnostic Risk Calculator for the Automatic Detection of Individuals at Risk and the Prediction of Psychosis: Second Replication in an Independent National Health Service Trust. *Schizophr Bull* 04 2019;45(3):562-570.

**34.** Koutsouleris N, Meisenzahl EM, Davatzikos C, et al. Use of Neuroanatomical Pattern Classification to Identify Subjects in At-Risk Mental States of Psychosis and Predict Disease Transition. *Archives of General Psychiatry* Jul 2009;66(7):700-712.

**35.** de Wit S, Ziermans TB, Nieuwenhuis M, Schothorst PF, van Engeland H, Kahn RS, Durston S, Schnack HG. Individual prediction of long-term outcome in adolescents at ultra-high risk for psychosis: Applying machine learning techniques to brain imaging data. *Human Brain Mapping* Feb 2017;38(2):704-714.

**36.** Koutsouleris N, Kambeitz-Ilankovic L, Ruhrmann S, et al. Prediction Models of Functional Outcomes for Individuals in the Clinical High-Risk State for Psychosis or With Recent-Onset Depression A Multimodal, Multisite Machine Learning Analysis. *Jama Psychiatry* Nov 2018;75(11):1156-1172.

**37.** Addington DE, Beck C, Wang JL, Adams B, Pryce C, Zhu HF, Kang J, McKenzie E. Predictors of Admission in First-Episode Psychosis: Developing a Risk Adjustment Model for Service Comparisons. *Psychiatric Services* May 2010;61(5):483-488.

**38.** Leighton SP, Krishnadas R, Chung K, et al. Predicting one-year outcome in first episode psychosis using machine learning. *Plos One* Mar 2019;14(3).

**39.** Fond G, Bulzacka E, Boucekine M, et al. Machine learning for predicting psychotic relapse at 2 years in schizophrenia in the national FACE-SZ cohort. *Prog Neuropsychopharmacol Biol Psychiatry* 06 2019;92:8-18.

**40.** Austin PC, Newman A, Kurdyak PA. Using the Johns Hopkins Aggregated Diagnosis Groups (ADGs) to predict mortality in a population-based cohort of adults with schizophrenia in Ontario, Canada. *Psychiatry Research* Mar 2012;196(1):32-37.

**41.** Bellon JA, Luna JD, King M, et al. Predicting the onset of major depression in primary care: international validation of a risk prediction algorithm from Spain. *Psychological Medicine* Oct 2011;41(10):2075-2088.

**42.** King M, Walker C, Levy G, et al. Development and Validation of an International Risk Prediction Algorithm for Episodes of Major Depression in General Practice Attendees The PredictD Study. *Archives of General Psychiatry* Dec 2008;65(12):1368-1376.

**43.** Nigatu YT, Liu Y, Wang JL. External validation of the international risk prediction algorithm for major depressive episode in the US general population: the PredictD-US study. *Bmc Psychiatry* Jul 2016;16.

**44.** Maarsingh OR, Heymans MW, Verhaak PF, Penninx B, Comijs HC. Development and external validation of a prediction rule for an unfavorable course of late-life depression: A multicenter cohort study. *Journal of Affective Disorders* Aug 2018;235:105-113.

**45.** Dinga R, Marquand AF, Veltman DJ, Beekman ATF, Schoevers RA, van Hemert AM, Penninx B, Schmaal L. Predicting the naturalistic course of depression from a wide range of clinical, psychological, and biological data: a machine learning approach. *Translational Psychiatry* Nov 2018;8.

**46.** Rubenstein LV, Rayburn NR, Keeler EB, Ford DE, Rost KM, Sherbourne CD. Predicting outcomes of primary care patients with major depression: development of a depression prognosis index. *Psychiatr Serv* Aug 2007;58(8):1049-1056.

**47.** Wang JL, Patten S, Sareen J, Bolton J, Schmitz N, MacQueen G. DEVELOPMENT AND VALIDATION OF A PREDICTION ALGORITHM FOR USE BY HEALTH PROFESSIONALS IN PREDICTION OF RECURRENCE OF MAJOR DEPRESSION. *Depression and Anxiety* May 2014;31(5):451-457.

**48.** Klein NS, Holtman GA, Bockting CLH, Heymans MW, Burge H. Development and validation of a clinical prediction tool to estimate the individual risk of depressive relapse or recurrence in individuals with recurrent depression. *Journal of Psychiatric Research* Sep 2018;104:1-7.

**49.** Hafeman DM, Merranko J, Goldstein TR, et al. Assessment of a Person-Level Risk Calculator to Predict New-Onset Bipolar Spectrum Disorder in Youth at Familial Risk. *JAMA Psychiatry* 08 2017;74(8):841-847.

**50.** Bauer IE, Suchting R, Van Rheenen TE, Wu MJ, Mwangi B, Spiker D, Zunta-Soares GB, Soares JC. The use of component-wise gradient boosting to assess the possible role of cognitive measures as markers of vulnerability to pediatric bipolar disorder. *Cognitive Neuropsychiatry* Mar 2019;24(2):93-107.

**51.** Ryu S, Lee H, Lee DK, Park K. Use of a Machine Learning Algorithm to Predict Individuals with Suicide Ideation in the General Population. *Psychiatry Investigation* Nov 2018;15(11):1030-1036.

**52.** Liu Y, Sareen J, Bolton JM, Wang JL. Development and validation of a risk prediction algorithm for the recurrence of suicidal ideation among general population with low mood. *Journal of Affective Disorders* Mar 2016;193:11-17.

**53.** Simon GE, Johnson E, Lawrence JM, et al. Predicting Suicide Attempts and Suicide Deaths Following Outpatient Visits Using Electronic Health Records. *American Journal of Psychiatry* Oct 2018;175(10):951-960.

**54.** Walsh CG, Ribeiro JD, Franklin JC. Predicting suicide attempts in adolescents with longitudinal clinical data and machine learning. *Journal of Child Psychology and Psychiatry* Dec 2018;59(12):1261-1270.

**55.** Tran T, Luo W, Phung D, Harvey R, Berk M, Kennedy RL, Venkatesh S. Risk stratification using data from electronic medical records better predicts suicide risks than clinician assessments. *Bmc Psychiatry* Mar 2014;14.

**56.** Kessler RC, Warner CH, Ivany C, et al. Predicting Suicides After Psychiatric Hospitalization in US Army Soldiers The Army Study to Assess Risk and Resilience in Servicemembers (Army STARRS). *Jama Psychiatry* Jan 2015;72(1):49-57.

**57.** Russo J, Katon W, Zatzick D. The development of a population-based automated screening procedure for PTSD in acutely injured hospitalized trauma survivors. *Gen Hosp Psychiatry* 2013 Sep-Oct 2013;35(5):485-491.

**58.** Papini S, Pisner D, Shumake J, Powers MB, Beevers CG, Rainey EE, Smits JAJ, Warren AM. Ensemble machine learning prediction of posttraumatic stress disorder screening status after emergency room hospitalization. *Journal of Anxiety Disorders* Dec 2018;60:35-42.

**59.** Rosellini AJ, Dussaillant F, Zubizarreta JR, Kessler RC, Rose S. Predicting posttraumatic stress disorder following a natural disaster. *Journal of Psychiatric Research* Jan 2018;96:15-22.

**60.** Galatzer-Levy IR, Ma S, Statnikov A, Yehuda R, Shalev AY. Utilization of machine learning for prediction of post-traumatic stress: a re-examination of cortisol in the prediction and pathways to non-remitting PTSD. *Transl Psychiatry* 03 2017;7(3):e0.

**61.** Karstoft KI, Galatzer-Levy IR, Statnikov A, Li ZG, Shalev AY, Jerusalem Trauma O. Bridging a translational gap: using machine learning to improve the prediction of PTSD. *Bmc Psychiatry* Mar 2015;15.

**62.** Galatzer-Levy IR, Karstoft KI, Statnikov A, Shalev AY. Quantitative forecasting of PTSD from early trauma responses: A Machine Learning application. *Journal of Psychiatric Research* Dec 2014;59:68-76.

**63.** Karstoft KI, Statnikov A, Andersen SB, Madsen T, Galatzer-Levy IR. Early identification of posttraumatic stress following military deployment: Application of machine learning methods to a prospective study of Danish soldiers. *Journal of Affective Disorders* Sep 2015;184:170-175.

**64.** King M, Bottomley C, Bellon-Saameno JA, et al. An international risk prediction algorithm for the onset of generalized anxiety and panic syndromes in general practice attendees: predictA. *Psychological Medicine* Aug 2011;41(8):1625-1639.

**65.** Nigatu YT, Wang JL. External validation of the International Risk Prediction Algorithm for the onset of generalized anxiety and/or panic syndromes (The Predict A) in the US general population. *Journal of Anxiety Disorders* May 2019;64:40-44.

**66.** Liu Y, Sareen J, Bolton J, Wang JL. DEVELOPMENT AND VALIDATION OF A RISK-PREDICTION ALGORITHM FOR THE RECURRENCE OF PANIC DISORDER. *Depression and Anxiety* May 2015;32(5):341-348.

**67.** Ngo DA, Rege SV, Ait-Daoud N, Holstege CP. Development and validation of a risk predictive model for student harmful drinking-A longitudinal data linkage study. *Drug and Alcohol Dependence* Apr 2019;197:102-107.

**68.** Afzali MH, Sunderland M, Stewart S, Masse B, Seguin J, Newton N, Teesson M, Conrod P. Machine-learning prediction of adolescent alcohol use: a cross-study, cross-cultural validation. *Addiction* Apr 2019;114(4):662-671.

**69.** Gueorguieva R, Wu R, O'Connor PG, Weisner C, Fucito LM, Hoffmann S, Mann K, O'Malley SS. Predictors of abstinence from heavy drinking during treatment in COMBINE and external validation in PREDICT. *Alcohol Clin Exp Res* Oct 2014;38(10):2647-2656.

**70.** Gueorguieva R, Wu R, Fucito LM, O'Malley SS. Predictors of Abstinence From Heavy Drinking During Follow-Up in COMBINE. *J Stud Alcohol Drugs* Nov 2015;76(6):935-941.

**71.** Hickey N, Yang M, Coid J. The development of the Medium Security Recidivism Assessment Guide (MSRAG): an actuarial risk prediction instrument. *Journal of Forensic Psychiatry & Psychology* 2009;20(2):202-224.

**72.** Hotzy F, Theodoridou A, Hoff P, Schneeberger AR, Seifritz E, Olbrich S, Jager M. Machine Learning: An Approach in Identifying Risk Factors for Coercion Compared to Binary Logistic Regression. *Frontiers in Psychiatry* Jun 2018;9.

**73.** Muñoz MA, Jeon N, Staley B, Henriksen C, Xu D, Weberpals J, Winterstein AG. Predicting medication-associated altered mental status in hospitalized patients: Development and validation of a risk model. *Am J Health Syst Pharm* Jun 2019;76(13):953-963.

**74.** Fernandez A, Salvador-Carulla L, Choi I, Calvo R, Harvey SB, Glozier N. Development and validation of a prediction algorithm for the onset of common mental disorders in a working population. *Aust N Z J Psychiatry* 01 2018;52(1):47-58.

**75.** Barker LC, Gruneir A, Fung K, et al. Predicting psychiatric readmission: sex-specific models to predict 30-day readmission following acute psychiatric hospitalization. *Social Psychiatry and Psychiatric Epidemiology* Feb 2018;53(2):139-149.

**76.** Fazel S, Wolf A, Larsson H, Lichtenstein P, Mallett S, Fanshawe TR. Identification of low risk of violent crime in severe mental illness with a clinical prediction tool (Oxford Mental Illness and Violence tool [OxMIV]): a derivation and validation study. *Lancet Psychiatry* 06 2017;4(6):461-468.

**77.** Chekroud AM, Zotti RJ, Shehzad Z, et al. Cross-trial prediction of treatment outcome in depression: a machine learning approach. *Lancet Psychiatry* Mar 2016;3(3):243-250.

**78.** Furukawa TA, Kato T, Shinagawa Y, et al. Prediction of remission in pharmacotherapy of untreated major depression: development and validation of multivariable prediction models. *Psychol Med* Oct 2019;49(14):2405-2413.

**79.** Maciukiewicz M, Marshe VS, Hauschild AC, et al. GWAS-based machine learning approach to predict duloxetine response in major depressive disorder. *Journal of Psychiatric Research* Apr 2018;99:62-68.

**80.** Serretti A, Olgiati P, Liebman MN, Hu H, Zhang Y, Zanardi R, Colombo C, Smeraldi E. Clinical prediction of antidepressant response in mood disorders: Linear multivariate vs. neural network models. *Psychiatry Research* Aug 2007;152(2-3):223-231.

**81.** Perlis RH. A Clinical Risk Stratification Tool for Predicting Treatment Resistance in Major Depressive Disorder. *Biological Psychiatry* Jul 2013;74(1):7-14.

**82.** Kautzky A, Dold M, Bartova L, et al. Clinical factors predicting treatment resistant depression: affirmative results from the European multicenter study. *Acta Psychiatrica Scandinavica* Jan 2019;139(1):78-88.

**83.** Kautzky A, Dold M, Bartova L, et al. Refining Prediction in Treatment-Resistant Depression: Results of Machine Learning Analyses in the TRD III Sample. *Journal of Clinical Psychiatry* Jan-Feb 2018;79(1).

**84.** Koutsouleris N, Kahn RS, Chekroud AM, et al. Multisite prediction of 4-week and 52-week treatment outcomes in patients with first-episode psychosis: a machine learning approach. *Lancet Psychiatry* Oct 2016;3(10):935-946.

**85.** Zandvakili A, Philip NS, Jones SR, Tyrka AR, Greenberg BD, Carpenter LL. Use of machine learning in predicting clinical response to transcranial magnetic stimulation in comorbid posttraumatic stress disorder and major depression: A resting state electroencephalography study. *Journal of Affective Disorders* Jun 2019;252:47-54.

**86.** Koutsouleris N, Wobrock T, Guse B, et al. Predicting Response to Repetitive Transcranial Magnetic Stimulation in Patients With Schizophrenia Using Structural Magnetic Resonance Imaging: A Multisite Machine Learning Analysis. *Schizophrenia Bulletin* Sep 2018;44(5):1021-1034.

**87.** Erguzel TT, Ozekes S, Gultekin S, Tarhan N, Sayar GH, Bayram A. Neural Network Based Response Prediction of rTMS in Major Depressive Disorder Using QEEG Cordance. *Psychiatry Investigation* Jan 2015;12(1):61-65.

**88.** Niles AN, Wolitzky-Taylor KB, Arch JJ, Craske MG. Applying a novel statistical method to advance the personalized treatment of anxiety disorders: A composite moderator of comparative drop-out from CBT and ACT. *Behaviour Research and Therapy* Apr 2017;91:13-23.

**89.** Bellou V, Belbasis L, Konstantinidis AK, Tzoulaki I, Evangelou E. Prognostic models for outcome prediction in patients with chronic obstructive pulmonary disease: systematic review and critical appraisal. *BMJ* 10 2019;367:l5358.

**90.** Moons KGM, Wolff RF, Riley RD, et al. PROBAST: A Tool to Assess Risk of Bias and Applicability of Prediction Model Studies: Explanation and Elaboration. *Ann Intern Med* 01 2019;170(1):W1-W33.

**91.** Royston P, Altman DG. External validation of a Cox prognostic model: principles and methods. *BMC Med Res Methodol* Mar 2013;13:33.

**92.** Kassraian-Fard P, Matthis C, Balsters JH, Maathuis MH, Wenderoth N. Promises, Pitfalls, and Basic Guidelines for Applying Machine Learning Classifiers to Psychiatric Imaging Data, with Autism as an Example. *Front Psychiatry* 2016;7:177.

**93.** Moons KG, de Groot JA, Bouwmeester W, Vergouwe Y, Mallett S, Altman DG, Reitsma JB, Collins GS. Critical appraisal and data extraction for systematic reviews of prediction modelling studies: the CHARMS checklist. *PLoS Med* Oct 2014;11(10):e1001744.

**94.** Wolff RF, Moons KGM, Riley RD, et al. PROBAST: A Tool to Assess the Risk of Bias and Applicability of Prediction Model Studies. *Ann Intern Med* 01 2019;170(1):51-58.

**95.** Christodoulou E, Ma J, Collins GS, Steyerberg EW, Verbakel JY, Van Calster B. A systematic review shows no performance benefit of machine learning over logistic regression for clinical prediction models. *J Clin Epidemiol* Jun 2019;110:12-22.

**96.** Lewis SJ, Arseneault L, Caspi A, et al. The epidemiology of trauma and post-traumatic stress disorder in a representative cohort of young people in England and Wales. *Lancet Psychiatry* Mar 2019;6(3):247-256.
